# Deep Learning Enhances Multiparametric Dynamic Volumetric Photoacoustic Computed Tomography In Vivo (DL‐PACT)

**DOI:** 10.1002/advs.202202089

**Published:** 2022-11-10

**Authors:** Seongwook Choi, Jinge Yang, Soo Young Lee, Jiwoong Kim, Jihye Lee, Won Jong Kim, Seungchul Lee, Chulhong Kim

**Affiliations:** ^1^ Department of Electrical Engineering Convergence IT Engineering Mechanical Engineering School of Interdisciplinary Bioscience and Bioengineering Graduate School of Artificial Intelligence and Medical Device Innovation Center Pohang University of Science and Technology (POSTECH) 77 Cheongam‐ro, Nam‐gu Pohang Gyeongbuk 37673 Republic of Korea; ^2^ Department of Chemistry POSTECH‐CATHOLIC Biomedical Engineering Institute Pohang University of Science and Technology (POSTECH) 77 Cheongam‐ro, Nam‐gu Pohang Gyeongbuk 37673 Republic of Korea

**Keywords:** deep learning, multiparametric imaging, photoacoustic imaging

## Abstract

Photoacoustic computed tomography (PACT) has become a premier preclinical and clinical imaging modality. Although PACT's image quality can be dramatically improved with a large number of ultrasound (US) transducer elements and associated multiplexed data acquisition systems, the associated high system cost and/or slow temporal resolution are significant problems. Here, a deep learning‐based approach is demonstrated that qualitatively and quantitively diminishes the limited‐view artifacts that reduce image quality and improves the slow temporal resolution. This deep learning‐enhanced multiparametric dynamic volumetric PACT approach, called DL‐PACT, requires only a clustered subset of many US transducer elements on the conventional multiparametric PACT. Using DL‐PACT, high‐quality static structural and dynamic contrast‐enhanced whole‐body images as well as dynamic functional brain images of live animals and humans are successfully acquired, all in a relatively fast and cost‐effective manner. It is believed that the strategy can significantly advance the use of PACT technology for preclinical and clinical applications such as neurology, cardiology, pharmacology, endocrinology, and oncology.

## Graphical abstract

The deep learning‐enhanced multiparametric dynamic volumetric PACT approach (DL‐PACT) improves the slow temporal resolution and at the same time qualitatively and quantitively diminishes the limited‐view artifacts that degrade image quality. DL‐PACT successfully performs high‐quality static structural and dynamic contrast‐enhanced whole‐body images as well as dynamic functional images of live animals and humans, all in a relatively fast and cost‐effective manner.

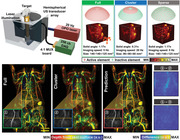

## 1 Introduction

Biomedical imaging modalities can provide comprehensive multiparametric information about complex biological systems in vivo.^[^ ^1^, ^2^ ^]^ In particular, by taking advantage of optical and ultrasound imaging technology, photoacoustic computed tomography (PACT) has become a premier biomedical imaging modality in both preclinical and clinical research.^[^ ^2^, ^3^, ^4^, ^5^, ^6^, ^7^, ^8^ ^]^ This hybrid imaging modality detects strong optical absorption contrast by exploiting the photoacoustic (PA) effect, which converts absorbed optical energy into acoustic waves.^[^ ^9^, ^10^, ^11^, ^12^ ^]^ Based on this fundamental principle, PACT provides structural, functional, and molecular information about animals and humans in vivo. The initial uses of PACT in preclinical and clinical research have expanded widely to include applications in cardiology,^[^ ^13^, ^14^ ^]^ neurology,^[^ ^9^, ^15^ ^]^ pharmacology,^[^ ^16^, ^17^, ^18^ ^]^ endocrinology,^[^ ^19^ ^]^ and oncology.^[^ ^20^, ^21^, ^22^, ^23^, ^24^, ^25^ ^]^

Because PA waves propagate omnidirectionally from a target, simultaneous and spherical collection of the PA signals around the target ideally avoids limited‐view artifacts and consequently attains excellent image quality.^[^ ^26^ ^]^ To meet this goal, a hemispherical detection scheme has been favored for premium PACT systems.^[^ ^7^, ^9^, ^15^, ^27^, ^28^, ^29^, ^30^, ^31^ ^]^ Once the PACT systems are combined with a hemispherical ultrasound (US) transducer array and multi‐channel data acquisition (DAQ) system, real‐time volumetric PACT systems can be designed, and further multiparametric dynamic imaging is feasible. A large aperture and a large number of elements in the hemispherical US transducer array further improve the image qualities, but a sufficient number of DAQ channels is also required for the fast‐imaging speed. Therefore, the system cost becomes quite high if one wants to achieve both excellent image qualities and fast temporal resolutions. Although multiplexing the PA signals can reduce the number of DAQ channels and the system cost, this approach slows the imaging speed.^[^ ^32^ ^]^

In this study, we propose a deep learning (DL) approach to solve the above‐mentioned problems in volumetric PACT. DL has emerged as a powerful tool to solve diverse problems in PA imaging, including removing image artifacts,^[^ ^33^, ^34^ ^]^ improving spatial resolution,^[^ ^35^, ^36^ ^]^ decreasing processing time,^[^ ^37^ ^]^ and reducing data throughput.^[^ ^38^, ^39^, ^40^ ^]^ Up until now, DL approaches have been explored in 2D PACT and have focused on enhancing structural image qualities. Here, for the first time, we demonstrate a DL‐enhanced multiparametric (i.e., morphological, functional, and contrast‐enhanced) dynamic volumetric PACT (DL‐PACT) system that qualitatively and quantitatively extends 3D deep neural networks (DNNs) to volumetric PACT.

Our DL strategy has the following advantages over previously reported approaches.^[^ ^38^, ^39^, ^40^ ^]^ First, we used only a quarter as many US elements in the hemispherical array (i.e., a cluster view) for the DNNs, but still produced high‐quality images that were comparable to images acquired with a full complement of US elements (full view). Further, this cluster view approach is more practical than using sparse data. US transducer arrays are typically manufactured by arranging the US elements in a sequential order, and thus it is difficult to apply a multiplexer that activates sparse channels at the same time. Therefore, although post‐DL processes can be employed with sparse data, a general multiplexing technique is not applicable. On the other hand, the cluster view can activate the element subset simultaneously through a general multiplexing technique, and then can accelerate the imaging speed. Moreover, our DL network is trained with PACT images of rats acquired at a particular wavelength, and it can subsequently be applied to images obtained at other wavelengths.

Based on this novel DL strategy, here we first demonstrate that the DL‐PACT can noninvasively acquire high‐quality static 3D whole‐body PACT images of live animals by overcoming the limited‐view aperture. Second, we confirm that contrast‐enhanced DL‐PACT can dynamically trace the pharmacokinetics of intravenously injected indocyanine green (ICG) at a high temporal resolution in vivo. Third, multi‐spectral DL‐PACT provides multiple functional parameters in live animals’ brains, such as the concentrations of oxy‐ (HbO) and deoxy‐hemoglobin (HbR), and the hemoglobin oxygen saturation (sO_2_). It provides this information quantitatively, dynamically, and rapidly. Furthermore, DL‐PACT measures accurate physiological phenomena with improved imaging speed and finally enhances the structural information of untrained subjects, such as tumor‐bearing mice and humans. We believe that our novel PACT strategy can significantly contribute to the widespread employment of PACT technology in a variety of preclinical and clinical applications, including neurology, cardiology, pharmacology, endocrinology, and oncology.

## 2 Results

### 2.1 DL‐Enhanced Multiparametric Dynamic Photoacoustic Computed Tomography (DL‐PACT) System

**Figure 1:**
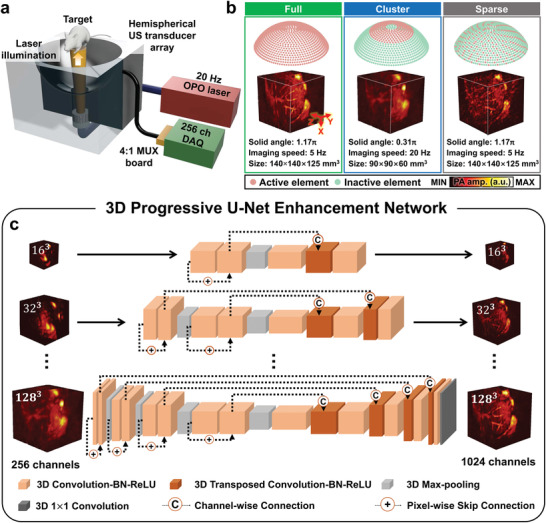
Deep‐learning‐enhanced multiparametric dynamic volumetric photoacoustic computed tomography (DL‐PACT) system and 3D progressive U‐net architecture. a) Schematic diagram of the DL‐PACT using a hemispherical US transducer array. b) Comparison of the arrangements of the transducer elements: full, cluster, and sparse views. c) Architecture of 3D progressive U‐net enhancement network. US, ultrasound; OPO, optical parametric oscillator; and MUX, multiplexer.

Our DL‐PACT system is based on a volumetric PACT using a 1024‐element hemispherical US transducer array (**Figure** **1**a and see Experimental Section; Figure S1, Supporting Information). An Nd:YAG pumped optical parametric oscillator (OPO) laser was used as an excitation source to provide laser illumination at a 20 Hz repetition rate in the near‐infrared spectrum, from 690–1064 nm. The OPO laser beam output was guided through a custom‐made fiber bundle and then inserted through the bottom of the transducer array. The imaging target was positioned at the center of the transducer array and moved for global raster scanning (Figure S1a, Supporting Information). Laser‐induced PA waves were received by the transducer array and then transferred to a 256 channels DAQ system after 4:1 multiplexing (MUX) board. Each US transducer element in the array had an average center frequency of 2.02 MHz and a bandwidth of 54% (Figure S1b, Supporting Information). The effective field‐of‐view (FOV) was 12.8 mm × 12.8 mm × 12.8 mm along x, y, and z axes, respectively, and the spatial resolutions of ≈380 µm were nearly isotropic along all directions when all 1024 US transducer elements were used (Figure S1c,d, Supporting Information).

With this system, we achieved 5‐Hz‐full‐view imaging with 1024 multiplexed US elements and 20‐Hz‐cluster‐view imaging with 256 non‐multiplexed ones (Figure 1b). The volumetric PA images from a rat's kidney shown in Figure 1b demonstrate the differences and trade‐offs in image quality. Compared to cluster‐view imaging, the full‐view imaging approach covers a larger solid angle (1.17*π* vs 0.31*π*) with a larger physical volume (140 mm × 140 mm × 125 mm vs 90 mm × 90 mm × 60 mm along x, y, and z axes, respectively). Full‐view imaging provides high‐quality PA images showing clear vasculatures and tissue boundaries, but it does so by sacrificing imaging speed (5 Hz for the full view vs 20 Hz for the cluster view). We further compared the system's performance with the sparse view, as reported in previous DL studies,^[^ ^38^, ^39^, ^40^ ^]^ with its performance using full and cluster views. Despite the streak artifacts in the sparse‐view image, fine structures are still visible in the sparse view because it has a larger solid angle than the cluster view. In addition, we cannot achieve to accelerate imaging speed with sparse view. Because like typical US transducer arrays, our transducer elements are arranged in a sequential order, we cannot activate the sparse subset simultaneously with the general MUX board.

A nearly isotropic resolution of ≈380 µm was achieved in both the full‐ and sparse‐view images, whereas in the cluster‐view image the XY plane resolution was ≈700 µm and the Z‐axis resolution was ≈300 µm (Figure S1e,f, Supporting Information). The effect of the limited‐view aperture is also obvious in the frequency domain (Figure S1g, Supporting Information), confirming that structures containing high spatial frequency information, such as sharp edges of organs and small vessels, are invisible in the cluster‐view image. It can be concluded that the full‐view approach can yield outstanding image qualities with high spatial resolution and a wide‐view aperture, whereas the cluster‐view mode can accelerate the imaging speed by four times, but with degraded image quality. To achieve both high temporal and spatial resolutions simultaneously without additional hardware (e.g., a massive multi‐channel DAQ system), we suggest a supervised deep learning approach in the following section.

### 2.2 3D Progressive U‐Net Architecture

We propose a 3D progressive U‐shaped enhancement network (3D‐pU‐net) to effectively process and learn volumetric PA representations from cluster‐ and full‐view imaging. The architecture of the proposed model is depicted in Figure 1c. The proposed model was constructed as a deep three‐dimensional convolutional neural network with an encoder–decoder structure, where the target volumetric data of the full‐view image is mapped using hierarchical spatial features extracted from the cluster‐view volumetric data. Inspired by recently developed progressive growth of generative adversarial networks (PGGAN) designed, the model was designed optimized to predict volumetric data via progressive increasing procedures.^[^ ^41^ ^]^ As visualized in Figure 1c, several sub‐networks are sequentially trained using downsampled data from the original high‐resolution volume data, and the obtained knowledge from each progressive step is gradually transferred. This progressive process enables the model to learn and reconstruct spatial characteristics at different scales, from the vasculatures to organs, by discovering structures in the coarser scale and detailed features in the finer scale. For training the neural network, we used 1089 single‐volume datasets with 128 × 128 × 128 voxels (a step size of 0.1 mm in all directions), acquired from 18 rats (see Experimental Section). We chose the voxel resolution of 128 × 128 × 128 to properly demonstrate the reconstruction capabilities of our DL model under maximum computational requirements for high‐resolution imaging. Additionally, as we mentioned, the spatial resolution is about 380 µm and the effective field of view is 12.8 mm × 12.8 mm × 12.8 mm. Thus, the voxel of 128 × 128 × 128 with a 0.1‐mm scale is enough to represent a single volume. We considered a multi‐part loss function combined with the voxel‐wise L1 loss and the 3D structural similarity (3D‐SSIM) index loss to describe the closeness between the full‐view images and the DL predictions (see Experimental Section). The data processing for the 3D‐pU‐net is detailed in Experimental Section and Figure S2 (Supporting Information). The prediction speed of the network is shown in Table S1 (Supporting Information). The best performance in generating a single volume is 0.016 seconds, which means we can practically utilize our network for real‐time dynamic imaging at up to 62.5 Hz.

### 2.3 DL‐Enhanced Static Whole‐Body PA Imaging of a Rat In Vivo

**Figure 2:**
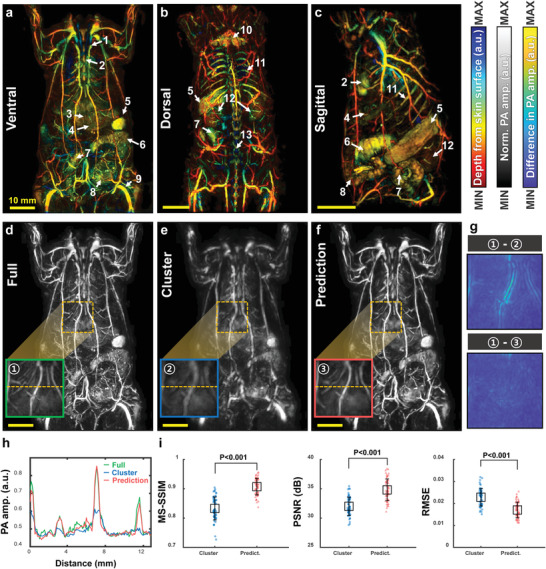
DL‐enhanced static whole‐body PA imaging of a rat in vivo. Whole‐body a) ventral, b) dorsal, and c) sagittal PA depth‐encoded images of a rat acquired with the full view (Movie S1, Supporting Information). Whole‐body ventral PA MAP images of a rat acquired with the d) full view, e) cluster view, and f) DL prediction (Movie S2, Supporting Information). Green, blue, and red boxes in (d–f) are close‐ups of single‐volume data from the orange dotted boxes in each image, respectively. g) PA images of the cluster‐view and prediction images subtracted from the full‐view image. h) Line profiles from the orange dotted lines in (d–f). i) Comparisons of the MS‐SSIM, PSNR, and RMSE of the cluster‐view and prediction images with respect to the full‐view images. All scale bars are 10 mm. Image labels: 1, sternum; 2, heart; 3, mesenteric artery; 4, liver; 5, spleen; 6, cecum; 7, small intestine; 8, large intestine; 9, popliteal vessel; 10, brown adipose tissue; 11, rib; 12, kidney; and 13, spine. MAP, maximum amplitude projection; MS‐SSIM, multi‐scale‐structural similarity index; PSNR, peak signal‐to‐noise ratio; and RMSE, root‐mean‐square error.

To investigate the imaging capability of the DL‐PACT, we acquired static whole‐body PA images of rats in vivo (**Figure** **2**). An optical wavelength of 900 nm was used, and these structural imaging results were used for the DL training process. Note that the reason we choose PA images at 900 nm for DL training is that they have better image quality and fewer artifacts compared to those at other wavelengths. Especially PA images at the near isosbestic point (e.g., 800 nm) have noisy artifacts appearing on the rat skin induced by depilatory (Figure S3, Supporting Information). Figure 2a–c and Movie S1 (Supporting Information) show the static whole‐body PA depth‐encoded images of the rat obtained with the full view in the ventral, dorsal, and sagittal planes, respectively. Major anatomical structures such as sternum (1), heart (2), liver (4), spleen (5), cecum (6), small intestine (7), large intestine (8), brown adipose tissue (10), rib (11), kidney (12), and spine (13) are clearly visualized. In addition, major vasculatures, such as mesenteric artery (3) and popliteal vessel (9) are apparently mapped. Next, the PA maximum amplitude projection (MAP) image from the full view was used as a ground truth (GT) and the subsampled data from the cluster view was used as the input for the 3D‐pU‐net (Figure S2, Supporting Information). Figure 2d–f and Movie S2 (Supporting Information) show the representative whole‐body ventral PA MAP images of the rat acquired with the full view, cluster view, and DL prediction, respectively, while the dorsal and sagittal images are shown in Figure S4 (Supporting Information). It is easily confirmed that the PA image with the full view (Figure 2d) is superior to that with the cluster view (Figure 2e), thanks to the wide aperture angle. More importantly, the DL prediction processed with the cluster‐view image (Figure 2f) significantly improves the image quality, making it very similar to the full‐view image, and well restoring the major organs (e.g., liver, intestines, and spleen) and vessels that are blurred in the cluster‐view image. Furthermore, from cross‐sectional images (Figure S5, Supporting Information), we can find that 3D‐pU‐net also recovered distorted vessels and organs along z‐axis and corrected depth information (Figure S6, Supporting Information). These results confirm that our 3D‐pU‐net works reliably with the DL‐PACT in all three geometrical dimensions.

To analyze our imaging results quantitatively, we zoom in on one single volume image (i.e., the green, blue, and red boxes in Figure 2d–f, number as 1, 2, and 3, respectively.). Figure 2g shows the results of subtracting the cluster‐view and prediction images, respectively, from the PA full‐view image, confirming that the DL prediction recovers the lost details in the cluster‐view image well. As shown in Figure 2h, the DL enhancement is further proved by comparing the line profiles cut along the orange lines in the green, blue, and red boxes in Figure 2d–f. Additional comparisons of other close‐up images in the dorsal and sagittal planes are shown in Figure S7 (Supporting Information). Figure S8 (Supporting Information) proves the DL‐enhanced spatial resolutions and the improved limited‐view aperture. Finally, we quantified three image quality metrics: the multi‐scale‐structural similarity index (MS‐SSIM), peak signal‐to‐noise ratio (PSNR), and root‐mean‐square error (RMSE) (Figure 2i). We extracted a total of 58 single‐volume images from one whole‐body ventral image, then compared one specific single‐volume‐full‐view image with the corresponding cluster‐view or DL‐predicted image to calculate the three parameters. The DNNs evidently improve the MS‐SSIM score from 0.83 ± 0.04 to 0.91 ± 0.03 (mean ± std.) and the PSNR from 32.0 ± 1.6 to 34.8 ± 1.9 dB, but suppress the RMSE from 0.025 ± 0.005 to 0.019 ± 0.004. Additional quality metric analyses from the dorsal and sagittal planes are shown in Figure S7 (Supporting Information). We also compared the performance of the proposed 3D‐pU‐net to that of the conventional 3D‐U‐net, which does not have progressive growth in the network (Figure S9a, Supporting Information). The 3D‐pU‐net's outputs and associated metrics are more comparable with the full‐view image than those of the conventional 3D‐U‐net (Figure S9b,c, Supporting Information).

### 2.4 DL‐Predicted Dynamic Contrast‐Enhanced Whole‐Body PA Imaging of a Rat Before and After an ICG Injection In Vivo

**Figure 3:**
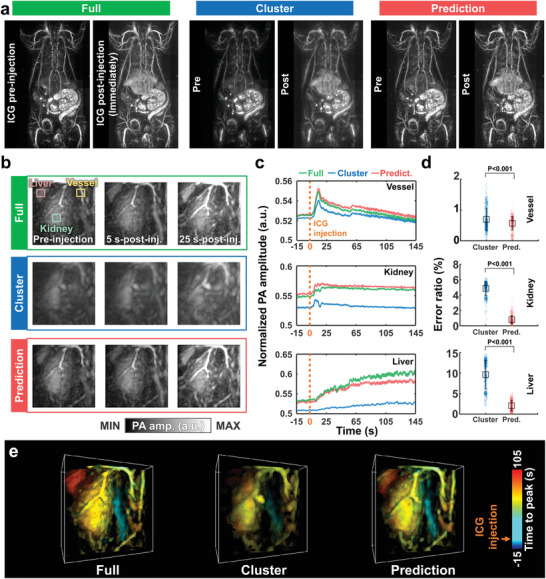
DL‐predicted dynamic contrast‐enhanced PA images. a) Whole‐body ventral PA MAP images of a rat in vivo before and after ICG injection. b) PA MAP images of the kidney in the full view, cluster view, and DL prediction (Movies S3, Supporting Information). c) Time‐lapse PA signals in the liver, kidney, and vessel. d) Comparisons of the error ratios of the cluster‐view and prediction images to the full‐view image in the vessel, kidney, and liver. e) 3D time‐to‐peak images of ICG perfusion in the kidney with the full view, cluster view, and DL prediction.

To explore the dynamic contrast‐enhanced imaging capability, we photoacoustically monitored the pharmacokinetics of intravenously injected ICG, an FDA‐approved contrast agent, in live animals.^[^ ^42^ ^]^ We acquired the dynamic contrast‐enhanced whole‐body PA images at an optical wavelength of 800 nm, but all of our DNN training was performed with the PA images obtained with an optical wavelength of 900 nm. In other words, the proposed 3D‐pU‐net is advantageous because it works independent of wavelength; otherwise, the training data for all‐optical wavelengths would have to be laboriously prepared. We also confirm this capability at wavelengths with distinct structural changes (i.e., 532, 660 nm) compared to the PA image at 900 nm in Figure S10 (Supporting Information). **Figure** **3**a shows contrast‐enhanced whole‐body PA images acquired before and after the ICG injection, with the full view, cluster view, and DL prediction. In contrast to the blurred‐cluster‐view images, the DL predicted images match well with the full‐view image both pre‐ and post‐injection. Soon after injection, PA signals within the major organs (e.g., liver, spleen, and bladder) and vessels significantly increase, and the time‐dependent DL predicted images agree well with the time‐course full‐view images (Figure S11, Supporting Information). Next, we dynamically traced the ICG kinetics in the vessel, kidney, and liver at 5 Hz of volumetric view before and after injection (Figure 3b). Note that the cluster‐view images are extracted from the full‐view images in post‐processing, meaning that the ground truth images (the full‐view images) always exist as references. Immediately after intravenous injection of ICG, the PA signal in the vessel increases significantly, followed by sequential increases in the kidney and liver. Moreover, the DNNs well recover the dynamic contrast‐enhanced PA image from the dimmed‐cluster‐view image. The ICG perfusion can be seen in Movie S3 (Supporting Information). The image‐by‐image comparison is performed at all time points, and the MS‐SSIM, PSNR, and RMSE are all statistically significantly improved with the DNNs (Figure S12, Supporting Information).

Figure 3c shows time‐variant changes in the PA signals within the vessel, kidney, and liver, quantified from the full‐view, cluster‐view, and DL‐predicted images. The DNNs consistently predict PA signals that are closer to those calculated from the full‐view image than those from the cluster‐view image. According to the Fourier spectrum analysis of the time‐sequential data, the rat's respiratory rate is accurately extracted from the DL prediction and full‐view images as 0.7 Hz, but the rate is not well recovered in some regions of cluster‐view image (Figure S13, Supporting Information). To quantitatively compare the time‐sequential data of the full view, cluster view, and DL prediction, we calculated an error ratio, defined as |*PA*_cluster or pred._ − *PA*_full_|/*PA*_full_, at a single time point. The calculated error ratios between the full view and the prediction are statistically significantly lower than those between the cluster and full views in all three regions (Figure 3d). Figure 3e shows time‐to‐peak volumetric PA images describing dynamic ICG perfusion, which again demonstrates the improved DL prediction capability.

Finally, as shown in Figure S14 and Movie S4 (Supporting Information), we acquired high‐quality dynamic contrast‐enhanced PA images at a frame rate of 20 Hz, the maximum imaging speed of our setup (i.e., the laser repetition rate). The imaging protocols were as follows: 1) For the first ICG injection, we obtained cluster‐view images in the kidney region at 20 Hz. 2) The DNNs generated the DL‐predicted images. 3) For the second ICG injection, full‐view images were acquired in the same region at 5 Hz as references, not as ground truths. The 20‐Hz‐predicted images well recover not only the main structures but also the dynamics. This result proves that our cluster‐view approach is more practical than the previously reported sparse‐view method.^[^ ^38^, ^39^, ^40^ ^]^ Note that the typical fabrication process of US transducer arrays places each element in sequential order, so it is impossible to activate randomly distributed sparse channels simultaneously using a typical multiplexer. Thus, the previous DL methods with sparse data are applicable only to post‐DL processes, not to improving the actual imaging speed. On the other hand, when a clustered subset in the US transducer array (the cluster view in our method) is used with the multiplexer, it is possible to activate all elements of the subset concurrently, allowing us to achieve the maximum imaging speed (the laser repetition rate in our case).

### 2.5 DL‐Enhanced Dynamic Functional PA Neuroimaging of a Rat In Vivo.

PACT is popular for label‐free functional neuroimaging of live animals because it provides the concentrations of oxy‐ (HbO) and deoxy‐hemoglobin (HbR), and consequently the total hemoglobin concentration (HbT) and hemoglobin oxygen saturation (sO_2_).^[^ ^6^, ^15^, ^43^ ^]^ To confirm DL‐enhanced PACT's label‐free functional imaging capability, we monitored the dynamic functional activities of rats during oxygen challenge tests.^[^ ^6^, ^42^, ^44^ ^]^ We alternated the inhaled gas concentrations between 90% oxygen/10% nitrogen (hyperoxia) and 10% oxygen/90% nitrogen (hypoxia) to induce changes in sO_2_. During the oxygen challenge test, we continuously and noninvasively acquired full‐view PA images of the rat's cortical region using two optical wavelengths, 750 and 850 nm. The cluster‐view image at each wavelength was extracted from the full‐view image, and the DL‐predicted image was then synthesized using the DNNs (see Experimental Section). Next, using the two‐wavelength PA images, a non‐negative spectral unmixing approach was used to determine the HbO, HbR, HbT, and sO_2_ (see Experimental Section).

**Figure 4:**
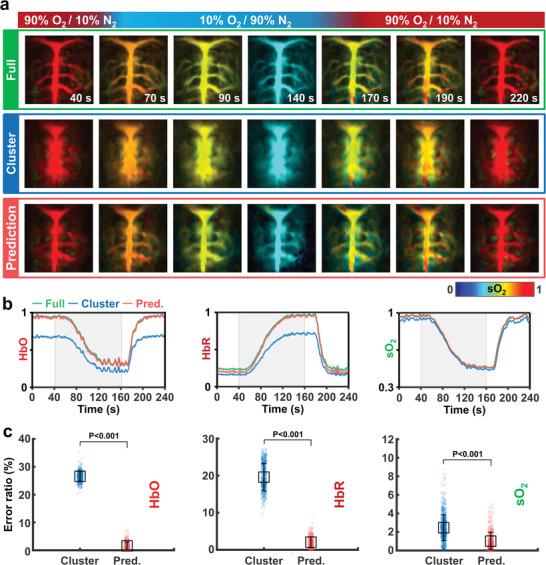
DL‐enhanced dynamic functional PA neuroimaging of a rat in vivo. a) Representative PA sO_2_ MAP images of a rat's brain in vivo, showing the full view, cluster view, and DL prediction under an oxygen challenge (Movie S5, Supporting Information). b) Time‐lapse PA HbO, HbR, and sO_2_ signals. c) Comparisons of the error ratios of the cluster‐view and prediction HbO, HbR, and sO_2_ images to the full‐view image. HbO, concentration of oxy‐hemoglobin; HbR, concentration of deoxy‐hemoglobin; and sO_2_, hemoglobin oxygen saturation.

**Figure** **4**a shows full‐view, cluster‐view, and DL prediction sO_2_ images of the cortical vessels during the oxygen challenge test at various time points. The whole process is dynamically visualized in Movie S5 (Supporting Information). In the full‐view data, major cerebral vessels are revealed with a high spatial resolution that aids in detecting their oxygenation state. However, except for the superior sagittal sinus in the center of the rat's brain, the blood vessels in the cluster‐view data are not clearly seen. Our 3D‐pU‐net successfully restores the missing details in the cluster‐view sO_2_ images and accords well with the full‐view images. Again, it is emphasized that our DNN training is not dependent on the excitation optical wavelength: The DNN training was performed at 900 nm, and the DNNs apply to the PA images acquired at 750 and 850 nm.

Figure 4b shows the time‐dependent functional parameters (HbR, HbO, and sO_2_) quantified from the full‐view, cluster‐view, and DL prediction images during the oxygen challenge. Even though the cluster‐views suffer from low image qualities, the overall changes in HbR, HbO, and sO_2_ from the cluster view closely follow the trends of those from the full‐view images. In particular, although the absolute HbR and HbO values in the cluster view deviate from those in the full view, the sO_2_ values are well correlated. More importantly, the DNNs quantitatively predict all three parameters well. Furthermore, comparing the DL‐predicted HbR, HbO, and sO_2_ images to the full‐view images, the error ratios are statistically significantly lower than those when comparing the cluster‐view images to the full‐view ones, again validating the superiority of our DNNs for functional neuroimaging (Figure 4c). Figure S15 (Supporting Information) shows additional quality metric comparison also proves the DL prediction superiorities and reliabilities.

### 2.6 DL‐Enhanced Physiological Signal Recording of a Rat In Vivo

**Figure 5:**
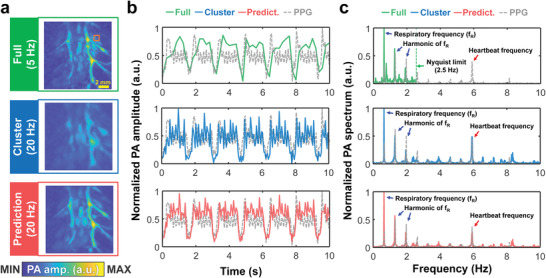
DL‐enhanced physiological signal recording of a rat in vivo. a) PA MAP images of the heart in the full view, cluster view, and DL prediction (Movie S6, Supporting Information) b) Time‐lapse PA signals and PPG signals in the heart. c) The power spectrum of the (b).

The PACT is a powerful tool to image and monitor the cardiac functions of murine models.^[^ ^6^, ^45^ ^]^ To investigate whether our DL model can recover the cardiac dynamics while preserving the image qualities, we obtained the PA images of rat hearts at 5 Hz (full view) and 20 Hz (cluster view) (**Figure** **5**a; Movie S6, Supporting Information). At the same time, we recorded physiological signals with a lab‐made photoplethysmography (PPG) at 20 Hz^[^ ^46^ ^]^ (see Experimental Section). Figure 5b,c shows the time‐lapse PA signals (from the orange box in Figure 5a) and PPG signals, and their associated frequency information, respectively. Compared with the PPG signals, the PA signals from the full‐view image could not monitor the full cardiac functions in both the time and frequency domains in spite of a good PA image quality. In contrast, the cardiac dynamics are fully recovered from the PA signals in the cluster‐view image, but the image quality is relatively poor due to the limited‐view effect. Obviously, the DL prediction is able to recover the full cardiac function while providing a good PA image quality, compatible with that from the full‐view image. It can be easily inferred that our DL method could potentially be a powerful tool for high‐resolution dynamic tracing applications such as tracking circulating tumor cells,^[^ ^6^ ^]^ super‐resolution localization imaging,^[^ ^36^ ^]^ and neuronal dynamics with a calcium indicator.^[^ ^15^ ^]^

### 2.7 DL‐Enhanced Static PA Imaging of Variable Subjects In Vivo

**Figure 6:**
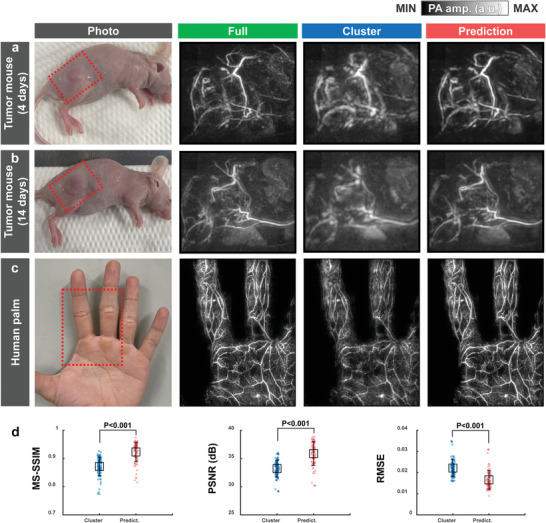
DL‐enhanced static PA imaging of variable subjects in vivo. PA MAP images of a tumor‐bearing mouse on the a) 4 and b) 14 days after 4T1 tumor cell injection in the full view, cluster view, and DL prediction. c) PA MAP images of a human palm in the full view, cluster view, and DL prediction. d) Comparisons of the MS‐SSIM, PSNR, and RMSE of the cluster‐view and prediction images with respect to the full‐view images.

To ensure the inter‐subject variability of our 3D‐pU‐net, we tested our DL model on other subjects such as tumor‐bearing mice and human palms. Note that all the training data sets are acquired from rats. We photoacoustically monitored the tumor progression on nude mice after subcutaneous injection of 4T1 tumor cells. The tumor area can be confirmed as shown in **Figure** **6**a. On the 14th post‐injection day, the tumor angiogenesis and tortuous microvessels are clearly visible (Figure 6b). While these complex vasculature changes are not obviously mapped in the cluster view data, the DL prediction better reconstructs the tumor region and well matches with the full‐view image. More interestingly, to demonstrate the DL‐PACT's capability of human imaging, we acquired PA images of a human palm (see Experimental Section). Although blood vessels are blurred and missing in the cluster‐view image, the DL prediction well recovers the details similar to them in the full‐view image (Figure 6c). The quantitative metrics also prove the superiority of our DL model (Figure 6d).

## 3 Discussion

PACT is a fast‐growing biomedical imaging modality that provides structural, functional, and molecular images of living subjects, with optical sensitivity and ultrasound resolution. Because PA waves propagate omnidirectionally, a hemispherical US transducer array is the best detector configuration for obtaining high‐quality PA images.^[^ ^13^, ^15^, ^27^ ^]^ A large number of elements and the large aperture of a hemispherical US transducer array improve the image qualities, but it is difficult to simultaneously achieve both high spatial and temporal resolutions, and such a system is expensive.

Here, we present a deep learning‐enhanced multiparametric dynamic volumetric PACT system (DL‐PACT) that solves the above‐mentioned problems. Recently, DL has been used to improve and enhance the performance of PACT systems in various ways, such as to improve spatial resolutions, enhance image quality, and reduce data throughput.^[^ ^33^, ^34^, ^35^, ^37^, ^38^, ^39^, ^40^, ^47^ ^]^ However, previous studies were mainly carried out with 2D PACT, and they concentrated on improving the quality of static images rather than imaging dynamic changes (e.g., functional activities or pharmacokinetics).^[^ ^34^, ^40^ ^]^ In addition, the strategies to reduce data throughput focused only on using sparse data, but it is practically difficult to activate the sparse channels simultaneously using a typical multiplexing board. In this study, by using a clustered dataset, we demonstrate that DL‐enhanced 3D PACT improves both the temporal and spatial resolutions. This strategy is successfully applied to dynamic contrast‐enhanced and functional imaging as well as static morphological imaging in live animals.

Our DL‐PACT utilizes a 3D progressive U‐net (3D‐pU‐net) to train 256‐element PACT images (referred to as the cluster view) with 1024‐element PACT images (referred to as the full view). This proposed ‘progressive’ approach can overcome the following limitations of the conventional 3D‐U‐net approach to predicting the high‐resolution full‐view images. First, the high‐resolution volumetric data representation of 128 × 128 × 128 voxels in the full and cluster views imposes an excessive amount of computational complexity on the model's learning process, making it difficult to achieve convergence of the learning process due to the lack of progressive processes. Second, from a loss minimization perspective, given that the loss values can be relatively small even if the predictions generally follow the ground‐truth volume images in a blurred form, the conventional 3D‐U‐net approach encounters difficulty in providing useful information for capturing fine details of volume images, and the learning process is likely to fall into the local minimum. In addition, the proposed approach is an advantageously versatile high‐resolution full‐view image reconstruction method. Our experimental results show that the proposed model, trained only with single‐wavelength data, can perform full‐view reconstruction on other wavelength data, demonstrating the scalability of the proposed approach for a variety of applications. Further, to our surprise, we demonstrate the sparse dataset can be applied to our 3D‐pU‐net trained by only the cluster‐view dataset (Figure S16, Supporting Information). Our DL approach can address two major challenges of the limited‐view effect and sparsity that occur when reducing the number of TR channels.

As the foundation of the whole work, we first test our framework in static imaging. The PA image quality from the cluster view is degraded significantly with blurred organs, distorted vessels, and lost detail structures, compared to the images from the full view. Our DL approach allows us to generate high‐resolution volumetric images that are comparable to the full view. All the imaging results (e.g., the depth‐encoded images, MAP images, cross‐section images, and single‐volume data) show that our DL approach successfully restores the structural information from the low‐quality images in the whole 3D view.

Observing dynamic contrast enhancement throughout the whole body plays an important role in pharmacokinetics.^[^ ^17^, ^22^ ^]^ We demonstrate the capability of DL‐PACT pharmacokinetic imaging with ICG injection. Our DL‐PACT predicts well the changes in PA signals, similar to the full‐view images without any extra training. When quantifying the PA signal change in the single volumetric view, the 3D‐pU‐net output images follow the full‐view data well over 1500 single frames with stable performance, while the cluster view shows the deviations in PA amplitudes. The high‐resolution images and accurate PA signal tracing enabled by 3D‐pU‐net help us to achieve time‐to‐peak images for ICG perfusion. These results indicate that 3D‐pU‐net output can serve as a reliable reference for monitoring dynamic changes in vivo. Besides, by performing the ground‐truth‐free DL‐PACT imaging at 20 Hz (the maximum imaging speed, limited by the laser repetition rate), we demonstrate that our DL‐PACT can track dynamic changes in organs accurately and efficiently, beyond the hardware limitations of the current system setup. In addition, we prove that our DL‐PACT can fully recover the cardiac dynamics (e.g., respiratory motion and heartbeats) while preserving the image qualities, and the results are validated via the PPG measurements.

Then, we show that DL‐PACT is effective for dynamic functional PA neuroimaging, which is one of PA imaging's key advantages. We perform dual‐wavelength (750 and 850 nm) PA imaging experiments to monitor the sO_2_ of a rat's brain during oxygen challenge. Spectrally unmixed data from two wavelengths synthesize the HbO, HbR, and sO_2_ images. To our surprise, the DL prediction shows constant performance with untrained two‐wavelength input, and the quantitative results of the 3D‐pU‐net are comparable with the full view.

Finally, we confirm that our DL model is immune to the imaging subjects through the inter‐subject variability test by imaging tumor‐bearing mice and human palms. The DL model well predicts the tumor progression and angiogenesis over time and rich vascular networks in human palms.

In conclusion, we prove that the DL‐PACT system overcomes the major intrinsic limitations of existing volumetric PACT systems, in which the hardware limits the image quality and imaging speed. We develop a volumetric PACT system using a hemispherical US transducer array to generate an outstanding high‐quality dataset and use an advanced progressive network for DL training. The DL‐PACT generates volumetric PACT images with solid angle coverage from 0.31*π* to 1.17*π* and improves the temporal resolution by four times. Further, the DL‐PACT is successfully applied to monitor intrinsic and extrinsic dynamics in vivo, which has not been achieved in other deep learning‐based PACT studies.

The capability of our DL‐PACT can be improved and expanded by addressing the following issues. First, the current full‐view PA image data has an inherent motion artifact caused by heartbeats, because the 256‐channel DAQ obtains the 1024‐element data with four laser pulses. Although the respiration artifacts are removed during the post‐image processing, the much faster heartbeat artifacts still exist. To fully acquire absolute ground truth images within the heart, either more DAQ channels or a higher repetition rate laser system is required. Second, model‐based reconstruction with the optimized speed of sound can further improve the image quality from the full‐view data, and then the DL prediction results can be further improved.^[^ ^47^, ^48^ ^]^ Third, although we address the limited‐view problem in the PACT, the limited‐bandwidth problem still remains. Our supervised DL approach has the potential to solve the limited‐bandwidth issue if we are able to acquire adapted broadband ground truth images.

For the first time, our DL‐PACT opens the possibility of enhancing all the features (static, dynamic contrast‐enhanced, and dynamic functional imaging) of volumetric PACT with a deep learning approach. In the future, we plan to target more humans and demonstrate the usability of clinical applications. We envision that DL‐PACT become much more commonly used as a major biomedical imaging modality in various preclinical and clinical applications.

## 4 Experimental Section

### Volumetric Photoacoustic Computed Tomography System and Performance Test

The main components of the volumetric PACT system were a hemispherical US array transducer, a DAQ system with a host computer, an OPO laser system, and a three‐axis motorized stage (Figure 1a; Figure S1a, Supporting Information). A customized 1024‐element hemispherical US transducer array was employed (Japan Probe, Inc., Japan; 60 mm radius; 2.02 MHz central frequency; 54% bandwidth). Each individual US element had an approximate area of 12 mm^2^. A high‐energy, mobile, and tunable laser system (PhotoSonus M‐20, Ekspla, Inc, Lithuania) was used as a PA excitation source. The laser system had a 3–5 ns pulse duration with a 20 Hz repetition rate. The output wavelength ranged from 690–1064 nm, with a maximum output pulse energy of 100 mJ. The laser beam was guided with a custom‐made fiber bundle (Opotek, Inc., USA) inserted through a hole in the center of the US transducer array. A pyroelectric energy sensor (ES220C, Thorlabs, Inc., USA) was used to record each laser pulse energy at the imaging plane. The pulse energies per unit area on the skin surface were 24.0 mJ cm^−2^ (750 nm), 22.7 mJ cm^−2^ (800 nm), 17.9 mJ cm^−2^ (850 nm), and 13.9 mJ cm^−2^ (900 nm), which were all well below the American National Standards Institute (ANSI) safety limits. The DAQ system (Vantage 256, Verasonics, Inc., USA) received the PA signals, digitizing them with programmable amplification up to 54 dB, triggered by the Q‐switch output of the laser system, and the sampling frequency was 8.33 MHz. To match acoustic impedance between the target and US array, animals were immersed in a custom‐made water tank. Note that only the animal's trunk was coupled with water, not the entire body. A water circulator (C‐WBL, Changshin Science, Republic of Korea) was used to control the water temperature in the water tank. A three‐axis gantry system (LSQ150A, Zaber, Inc., USA) was used to perform raster scanning for whole‐body imaging by moving the target in the water tank as shown in Figure S1a (Supporting Information). To benchmark the system performance, volumetric images of microspheres were obtained with diameters of about 50 µm (BKPMS‐1.2 45–54 µm, Cospheric, USA) embedded in agarose gel phantoms (Figure S1c, Supporting Information).

### PA Image Reconstruction

A delay‐and‐sum algorithm, accelerated by parallel computing with CUDA, was used to reconstruct volumetric PA images. (Figure S2a, Supporting Information).^[^ ^49^ ^]^ The reconstruction was performed on a grid of 128 × 128 × 128 voxels (the voxel size was 0.1 mm in all directions) with a homogeneous speed of sound. A whole‐body image was synthesized by stitching together the single volumetric images acquired at each scanning position. There was an overlapped region between two adjacent single volumes to avoid seams. The voxel value of the intersection region of two adjacent single volumes was determined by the maximum compounding method.^[^ ^50^ ^]^ Depth‐encoded images were generated via 3DPHOVIS.^[^ ^51^ ^]^

### DL Data Preprocessing

Pairs of training datasets were prepared from the full‐view dataset (Figure S2b, Supporting Information). First, an individual full‐view image was reconstructed with the raw data acquired from the 1024 US elements. Those full‐view images served as ground truth images for training. Then the raw data were selected from the 1st to 256th channels and the cluster‐view image was reconstructed as the training input. These training datasets were normalized based on the maximum and minimum values of the whole‐body images. For deep learning network training, a total of 1089 PACT volume images were used: 1069 volumes from whole‐body scanning of 18 rats, 10 kidney‐specific volumes, and 10 brain‐specific volumes (Table S2, Supporting Information).

### Image Quality Metrics

The image quality of cluster‐view or prediction images (referred to as *I*) were evaluated by using full reference quality metrics with reference to corresponding ground truth images (here, the full view was referred to as *I* ^GT^). MS‐SSIM,^[^ ^52^ ^]^ PSNR, and RMSE were computed for all experimental results. The SSIM was a general reference metric of similarity between two images. This index considered the luminance and contrast as well as structural information, and it was defined as

(1) $$\mathrm{SSIM}\left(x,y\right)=\frac{\left(2\mu_{x}\mu_{y}+C_{1}\right)\left(2\sigma_{\mathrm{xy}}+C_{2}\right)}{\left(\mu_{x}^{2}+\mu_{y}^{2}+C_{1}\right)\left(\sigma_{x}^{2}+\sigma_{y}^{2}+C_{2}\right)}$$

where *μ*_*x*_ is the average of *x*, *σ*_*x*_ is the variance of *x*, and *σ*_*xy*_ is the covariance of *x* and *y*. The MS‐SSIM was computed by combining the SSIM index at various scales with the following values: NumScale = 4, Sigma = 8, and default ScaleWeight.

The PSNR is a metric that estimates discrepancies between two images with respect to the peak signal amplitude of the prediction image:

(2) $$\mathrm{PSNR}=10\log_{10}\left(\mathrm{peak}\mathrm{va}l^{2}/\mathrm{MSE}\right)$$

where *peakval* is taken from the range of the image data type.

The mean squared error (MSE) is defined as^[^ ^53^ ^]^

(3) $$\mathrm{MSE}=\frac{1}{N}\sum_{i}{\left(I-I^{\mathrm{GT}}\right)}^{2},\mathrm{RMSE}=\sqrt{\frac{1}{N}\sum_{i}{\left(I-I^{\mathrm{GT}}\right)}^{2}}$$

where *N* is the total number of voxels. RMSE is the square root of MSE. All those metrics are calculated using built‐in functions in MATLAB.

All data are presented as mean ± s.d. (standard deviation) or as individual plots. Statistic significances for two‐group comparisons were determined by two‐tailed Student's t‐tests. For multiple‐group comparisons, ANOVA tests were used. Statistical tests were carried out using GraphPad Prism (v7.0a) (GraphPad, USA) or MATLAB (R2021a) (MathWorks, USA).

### 3D Progressive U‐Net Architecture and Learning Scheme

A 3D progressive U‐shaped convolutional neural network (3D‐pU‐net) was proposed to accomplish high‐resolution volumetric PACT enhancement. The overall architecture of the proposed model was visualized in Figure 1c. The U‐shaped DNNs were mainly established with encoder–decoder structures trained to map the nonlinear relationship between the input and output of the developed PACT system. Further, several channel‐wise and voxel‐wise skip connection mechanisms were taken advantage of to encourage the model to reuse the extracted contextual information of the volume data and mitigate the vanishing gradient problem of the deeply constructed neural network, achieving better gradient flow in a backpropagation learning scheme. It is particularly noteworthy that the progressive framework was used to achieve high‐resolution enhancement, where the proposed model with several sub‐networks was trained to reconstruct finer details by appending additional layers for different scales of volume data. Inspired by the PGGAN,^[^ ^41^ ^]^ it is hypothesized that the delicate details from various imaging components (e.g., vessels and organs) can be reconstructed by gradually increasing the complexity of the enhancement network. In this way, the progressive framework leveraged its learning capability from the approximate shape at a coarser scale to the detailed shape at a finer scale. It employed an incremental learning procedure of enlarging spatial dimension in a progressive way, from low‐ to high‐resolution volumetric images.

The progressive learning process worked as follows. First, volume data with an ultimate resolution of 128 × 128 × 128 voxels were downscaled to several different scales, where four sub‐networks with increasingly larger scales, i.e., 16^3^, 32^3^, 64^3^, 128^3^, were consecutively trained. Their learned parameters were transferred to subsequent higher scale network until the final scale of the sub‐network was reached. To avoid abrupt changes in the trainable parameters of the preceding network, the smooth fade‐in technique was employed to gradually adapt the output layer driven by the previous sub‐network's parameters and the present sub‐network's newly learned parameters. Regarding the training set of the proposed model, 1089 volume data with 128^3^ voxels in a 0.1 mm cube were used. To optimize the dynamic output, 20 volumetric images from single organs, including the kidney (*n* = 10) and brain (*n* = 10), were used. The volume‐based multipart loss function was accounted for to measure the visual similarity of the 3D volume data, where the L1‐norm and 3D‐SSIM index were utilized to determine the spatial and structural resemblance between the full‐view and the predicted images. The combined loss function *J* could be expressed as

(4) $$J\left(V_{\mathrm{fv}},\hat{V_{\mathrm{fv}}}\right)=\lambda \times J_{L1}+\left(1-\lambda \right)\times \left(1-J_{3D-\mathrm{SSIM}}\right)$$

where *λ* controls significance between the L1‐norm difference *J*_L1_ and the 3D‐SSIM difference (1 − *J*_3D − SISM_), while a *λ* of 0.2 was used in this study.^[^ ^54^ ^]^ Through tuning the proposed model, it is empirically found that combining multiple loss functions could produce better reconstruction outcomes than employing a stand‐alone loss function (Results comparisons among stand‐alone and combined loss functions are provided in Figure S17, Supporting Information). Several previous studies had also revealed that adopting a single loss function, such as the pixel‐level L1/L2 or SSIM, has several drawbacks, including the lack of structural/perceptual reconstruction quality and high‐frequency details.^[^ ^54^, ^55^, ^56^, ^57^, ^58^, ^59^, ^60^ ^]^ While the combined loss function technique had been successfully utilized in several prior research,^[^ ^36^, ^55^, ^61^ ^]^ the analogous approach was also employed that could impose both pixel‐level and structural information on the model's learning process, aiming to achieve the plausible reconstruction of structures (such as vessels and organs) as well as individual signal amplitudes for applications ranging from static whole‐body imaging to dynamic functional imaging.

Training and regularization settings were given as in the following. In terms of the batch size in the mini‐batch gradient descent learning process, the batch size was varied in the order of 16, 16, 8, and 4 as the progressive learning of the sub‐network proceeds. A 3D batch normalization layer was appended to each 3D convolutional layer, where the momentum parameter was set to be 0.1 and *ε* was set to be 1e‐5. While the AdamOptimizer was utilized, a learning of 1e‐4 was assigned, which could be reduced via plateaus of the validation loss (ReduceLRonPlateau implemented in PyTorch). Besides, L2 regularization with its coefficient of 2e‐6 and an early‐stopping strategy during all 200 training epochs were considered for regularizing the training process. The detailed hyper‐parameter settings were in Table S3 (Supporting Information). The networks were implemented using a Python 3.7.8/PyTorch 1.9.0 framework and trained using an NVIDIA GeForce RTX 3090.

### Animal Preparation

All experimental procedures followed the regulations and guidelines approved by the Institutional Animal Care and Use Committee (IACUC) of Pohang University of Science and Technology (POSTECH). For all in vivo experiments except oncologic imaging, 3 or 4‐week‐old Sprague Dawley rats (Dawley SD, Harlan Co.; 80–100 g body weight) were used. Before the imaging experiments, the hair of the rat or mouse was removed using an electronic shaver and depilatory cream. During the whole‐body scan, the rat was secured in a lab‐made water tank with a transparent imaging window at the bottom. The rat was anesthetized with isoflurane using a gas system (VIP 3000 Veterinary Vaporizer, Midmark, USA). The rat's front teeth are made to bite a breathing mask to fix the rat's position and prevent it from becoming separated from the anesthesia system (Figure S1a, Supporting Information). The rat trunk was immersed while maintaining its body temperature by circulating the water through a heating bath outside the tank. For brain imaging, a custom‐made stereotaxic mount was used to fix the brain, and the cortical surface was positioned flat and lined up with the transducer's focal plane. In contrast‐enhanced imaging, 0.15 mL ICG solution (1 mg mL^−1^) was intravenously injected. For functional PA neuroimaging, the oxygen challenge protocol from a previous study was followed.^[^ ^42^ ^]^ A mixture of 90% oxygen/10% nitrogen was initially used for 2 min, then the mixture was changed to 10% oxygen/90% nitrogen for 2 min, and finally changed back to the initial concentration. The gas mixture was controlled manually using a multi‐gas flowmeter, keeping the isoflurane level constant in the inhaled gas. For the oncologic study, a female Balb/c‐nu/nu mouse (6 weeks old, 16 g), was provided from POSTECH Biotech Center (Pohang, Republic of Korea). 4T1 breast cancer cell line was obtained from the American Type Culture Collection (ATCC). 4T1 cells (Cat# CRL‐2539) were cultured on a T175 cell culture flask with RPMI 1640 media containing 10% fetal bovine serum (FBS), 100 U mL^−1^ penicillin (PS), and 100 µg mL^−1^ streptomycin and incubated in a 5% CO_2_ humidified incubator at 37 °C. 4T1 cells were inoculated subcutaneously at an initial density of 7×10^5^ cells per mouse in a 50/50 v/v mixture with BD Matrigel basement membrane matrix (BD Biosciences) into the right thigh of mice under isoflurane anesthesia. In vivo imaging experiment was performed when the tumor volume reached 60 mm^3^ on day 4 after inoculation. Then, when the tumor reached 300 mm^3^ at 14 days after injection, in vivo imaging was performed again. The tumor volume was calculated as (length)×(width)^2^/2. After all in vivo experiments, animals were sacrificed by CO_2_ asphyxiation according to an approved procedure.

### PA Imaging of Human Palms

All human experimental procedures followed a clinical protocol approved by the Institutional Review Board (IRB) of POSTECH (PIRB‐2022‐E‐26). One healthy volunteer as the subject was imaged in this study. The experimental procedure was explained and his informed consent was received before the experiment. The operator and subject wore laser safety glasses and flame‐retardant clothes for protection from the laser beam.

### Data Processing for Dynamic Contrast‐Enhanced and Functional Imaging Results

To visualize ICG perfusion, 1500 PA image frames were taken within the kidney region at 5 Hz. Then 800 frames were selected for quantitative analysis (Figure 3; Figure S12, Supporting Information) and 1500 frames were used for Movie S3 (Supporting Information). Three volumes‐of‐interest (VOI) with 10 × 10 × 10 voxels were manually selected at different positions to trace the ICG kinetics. The PA signal was recorded using the mean value of the VOI voxels in each frame, and the time‐lapse PA signal was then filtered with a 5‐frame moving average filter.

For functional PA neuroimaging, 1800 images were taken using the fast‐ wavelength‐tuning mode of the laser system. Before spectral unmixing, the negative values were removed and a 3D median filter (neighborhood size = [3 3 3]) was applied to the normalized PA images to smooth pixel‐wise mismatch. Then, linear least square fitting (LSQ) unmixing was applied on a per voxel basis, using the known molar extinction coefficient spectra of the main blood chromophores, the oxygenized (HbO), and the deoxygenized (HbR) as follows:^[^ ^19^ ^]^

(5) $$\left[\begin{array}{c} C_{\mathrm{HbO}}\\ C_{\mathrm{HbR}} \end{array}\right]={\left[\begin{array}{cc} \varepsilon_{\mathrm{HbO}}^{750} & \varepsilon_{\mathrm{HbR}}^{750}\\ \varepsilon_{\mathrm{HbO}}^{850} & \varepsilon_{\mathrm{HbR}}^{850} \end{array}\right]}^{-1}\left[\begin{array}{c} PA_{750}\\ PA_{850} \end{array}\right].$$

To calculate the sO_2_, we use the following equation:

(6) $$sO_{2}=\frac{C_{\mathrm{Hb}O_{2}}}{C_{\mathrm{Hb}}+C_{\mathrm{Hb}O_{2}}}.$$

In the above equation, PA represents the PA amplitude after the compensation process, *ε* represents the molar extinction coefficient, and *C* is the concentration. A volumetric mask corresponding to the total hemoglobin (HbR+HbO) was generated to separate vessel‐related voxels from background noise by using 3D PHOVIS. The mean values over the volumetric mask from the HbR, HbO, and sO_2_ values at single time points were filtered using the 5‐frame moving window, and then the values were plotted as a function of time to show the dynamic change. The sO_2_ MAP images were generated by the masked sO_2_ data.

For cardiac imaging, the PA images were obtained from the heart region at 5 Hz with the full view and at 20 Hz with the cluster view. The PPG signals were simultaneously recorded. One volumes‐of‐interest (VOI) with 10 × 10 × 10 voxels was selected for signal analysis at the right atrium based on the full‐view PA images. The time‐domain PA signal was transformed into the frequency domain by MATLAB built‐in functions, and then the respiratory and/or the heartbeat frequency components were extracted. The lab‐made PPG board composed of a light emitting diode (LED, VLMTG1400, Vishay Semiconductors, Malvern, PA, USA) with 532 nm and a silicon‐based photodiode (PD, SFH2716, Osram Opto Semiconductors, Regensburg, Germany).^[^ ^46^ ^]^

## Conflict of Interest

C.K. has financial interests in OPTICHO, which, however, did not support this work.

## Supporting information

Supporting Information

Supplemental Movie 1

Supplemental Movie 2

Supplemental Movie 3

Supplemental Movie 4

Supplemental Movie 5

Supplemental Movie 6

## Data Availability

The data that support the findings of this study are available from the corresponding author upon reasonable request.
