# Supplementary material for: Deep Learning Enhances Multiparametric Dynamic Volumetric Photoacoustic Computed Tomography In Vivo (DL‐PACT)
Source: Adv Sci (Weinh). 2022 Nov 10;10(1):2202089. doi: 10.1002/advs.202202089 (PMC9811490; doi:10.1002/advs.202202089)
Supplement: Supplementary file 1 — Supporting Information [file ADVS-10-2202089-s006.pdf]

Supporting Information

Deep Learning Enhances Multiparametric Dynamic Volumetric Photoacoustic Computed Tomography *in Vivo* (DL-PACT)

Seongwook Choi[1†], Jinge Yang[1†], Soo Young Lee[1†], Jiwoong Kim[1], Jihye Lee[2], Won Jong Kim[2], Seungchul Lee[1], and Chulhong Kim[1*]*

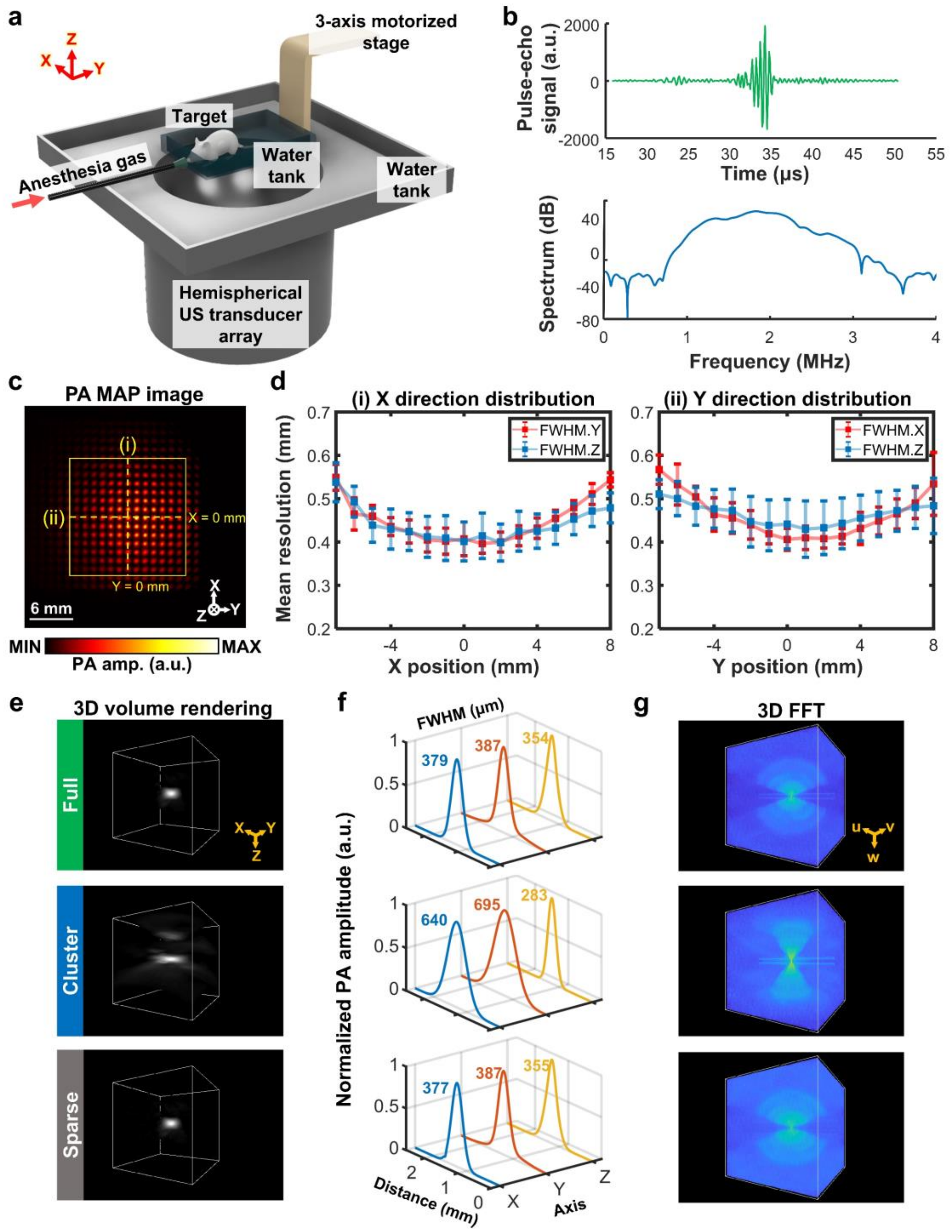

Figure S1.

(a) Schematic of the scanning system. **(b)** Time- and frequency-domain responses of one representative US element in the hemispherical array. **(c)** PA MAP image of microsphere targets. **(d)** Spatial resolutions along the x, y, and z axes. **(e)** 3D PA images of single microsphere. **(f)** Associated spatial resolutions along each axis, and **(g)** 3D FFT results in the full, cluster, and sparse views, respectively.

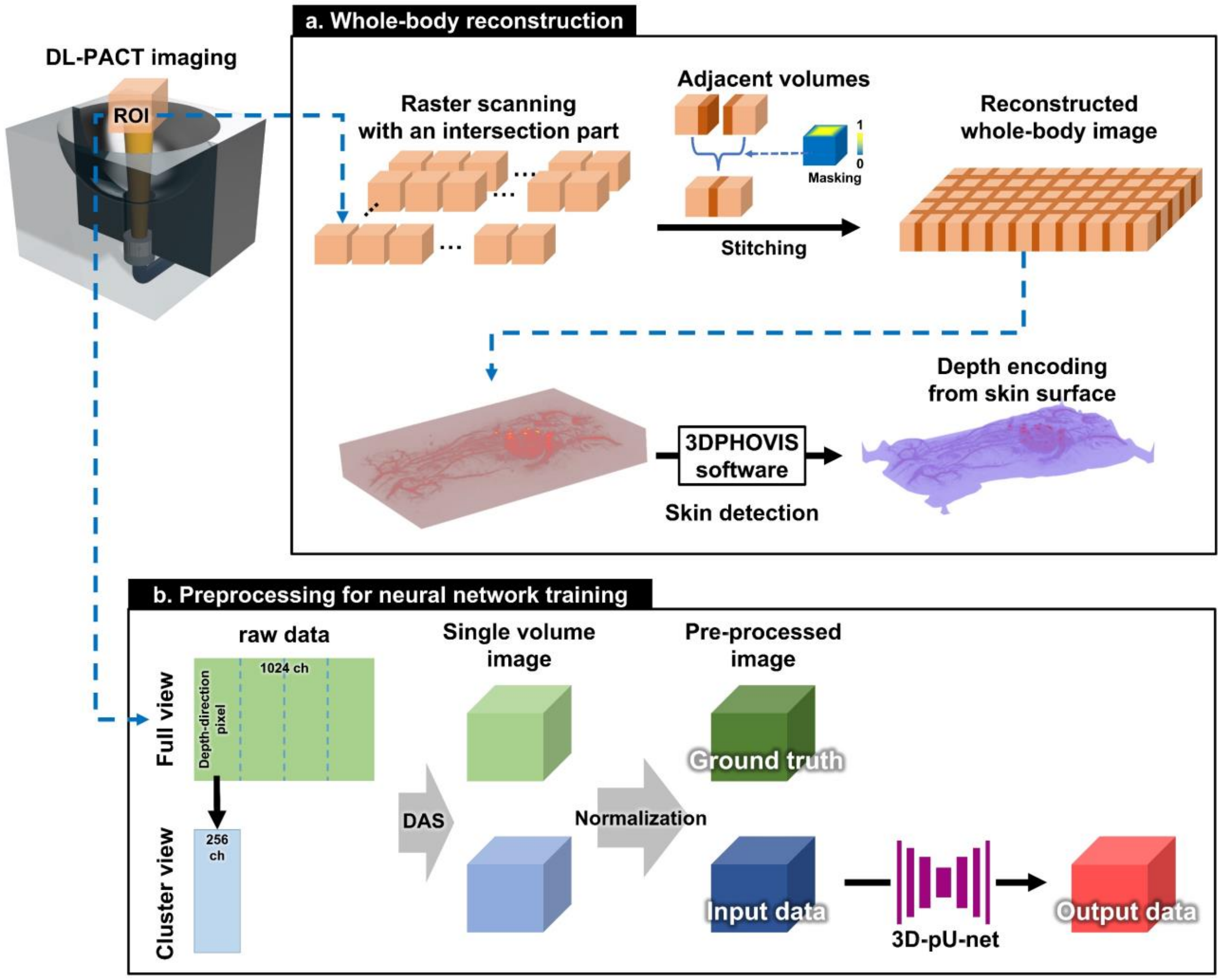

Figure S2.

(a) Schematic of whole-body image reconstruction process. **(b)** Schematic of preprocessing for training deep neural networks. DAS, delay and sum.

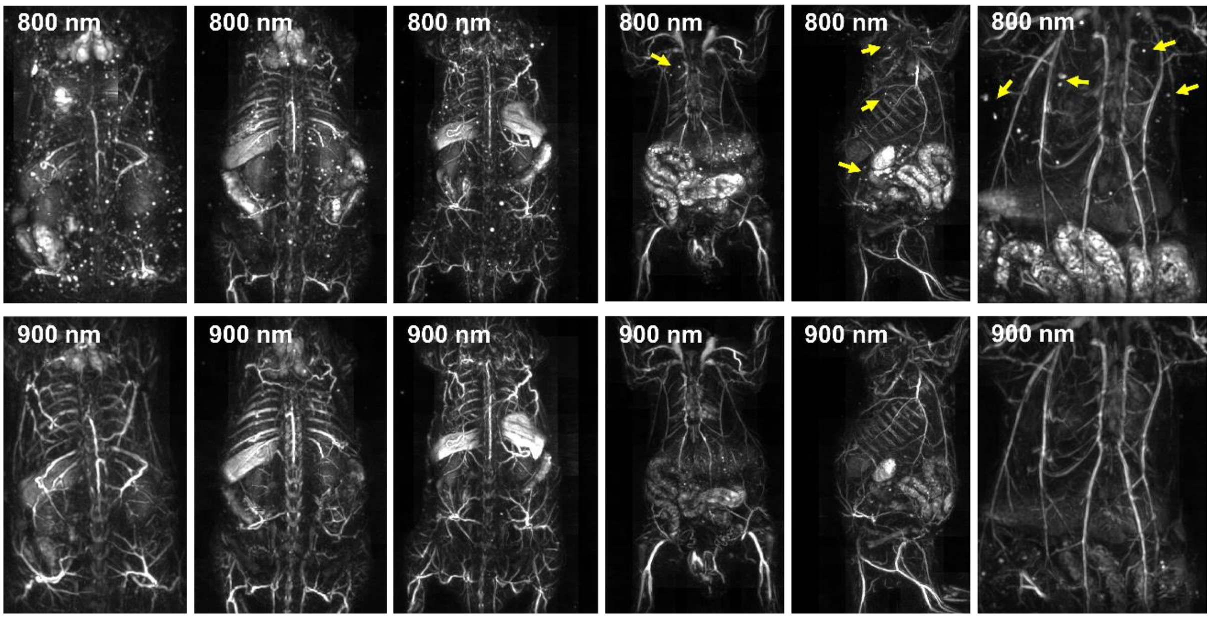

Figure S3.
Comparison of full-view PA MAP images acquired at 800 nm and 900 nm.

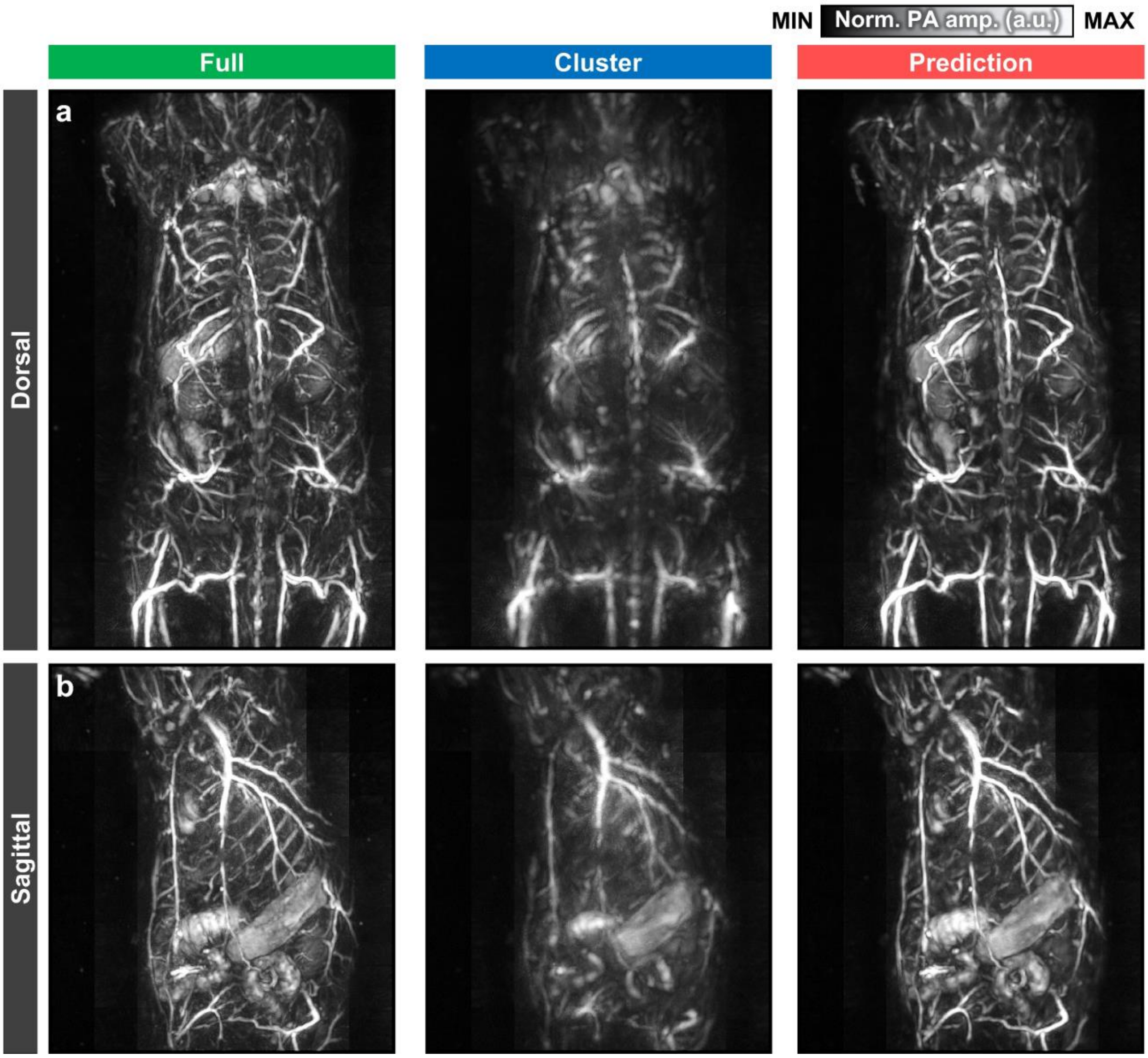

Figure S4.

(a) Whole-body dorsal PA MAP images of a rat *in vivo*: full view, cluster view, and DL prediction. **(b)** Whole-body sagittal PA MAP images of a rat *in vivo*: full view, cluster view, and DL prediction.

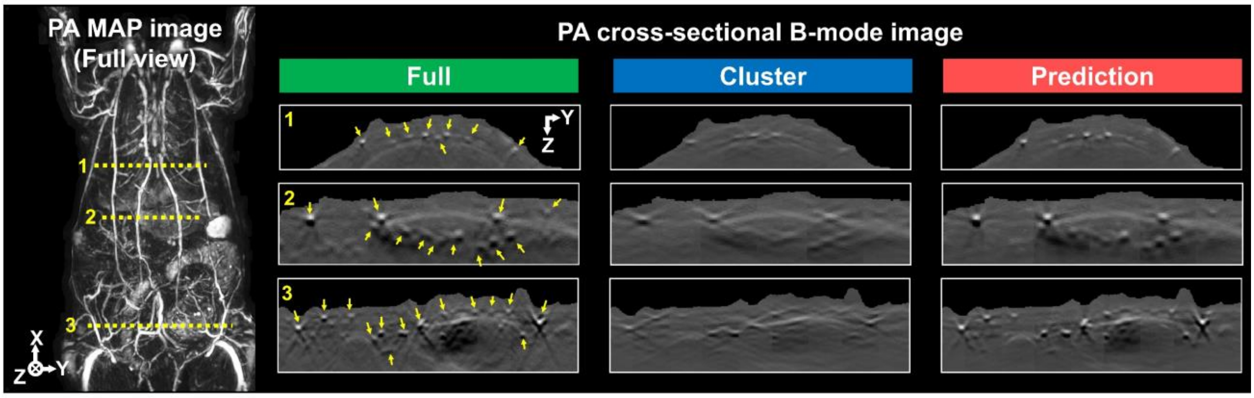

Figure S5.

Comparison of PA cross-sectional images cut along the yellow dotted lines: full view, cluster view, and DL prediction.

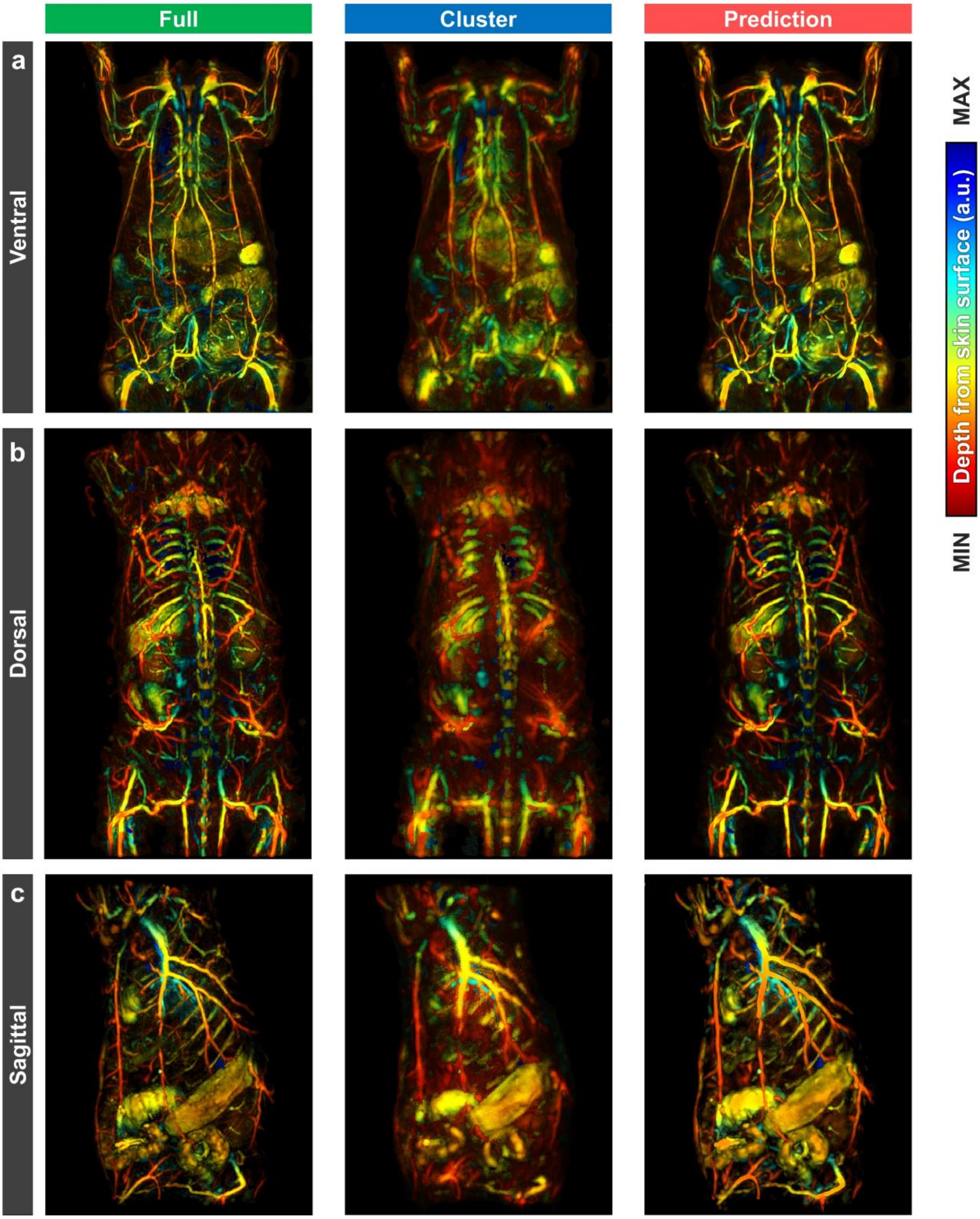

Figure S6.
Whole-body PA depth-encoded images of the rat: full view, cluster view, and DL prediction in the **(a)** ventral, **(b)** dorsal, and **(c)** sagittal planes *in vivo*.

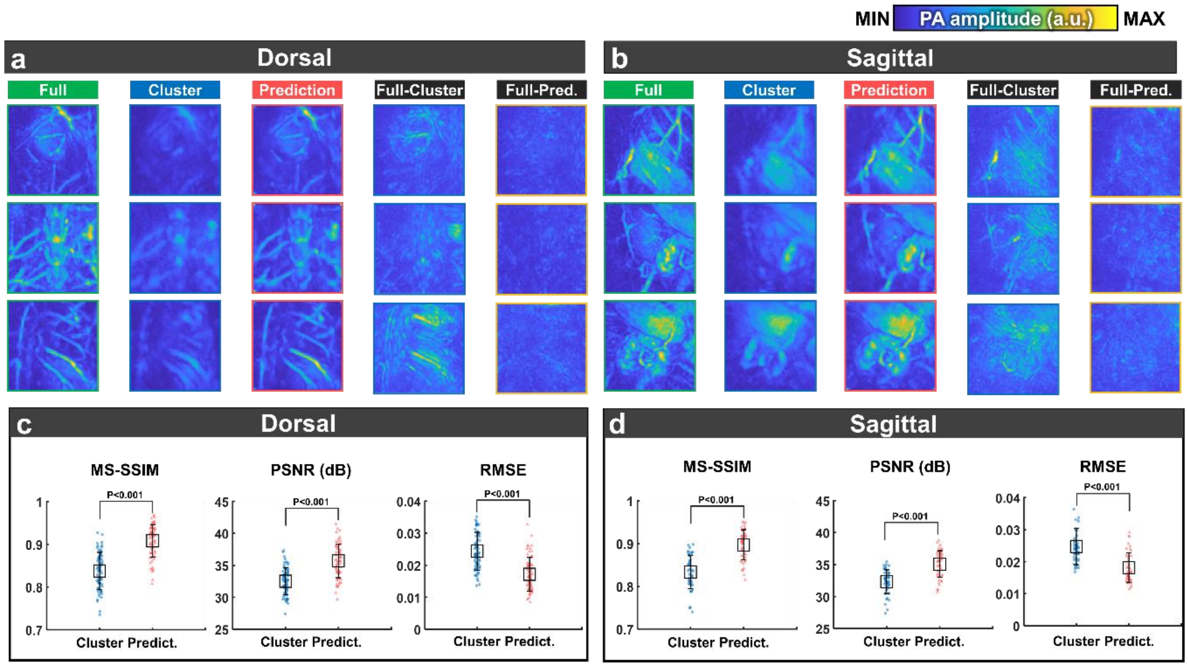

Figure S7.

PA MAP images of single-volume data and the subtraction images between the full-view image and cluster-view/prediction images from the **(a)** dorsal and **(b)** sagittal planes. Comparisons of the MS-SSIM, PSNR, and RMSE of the cluster-view and prediction images with respect to the full-view image in the **(c)** dorsal and **(d)** sagittal planes.

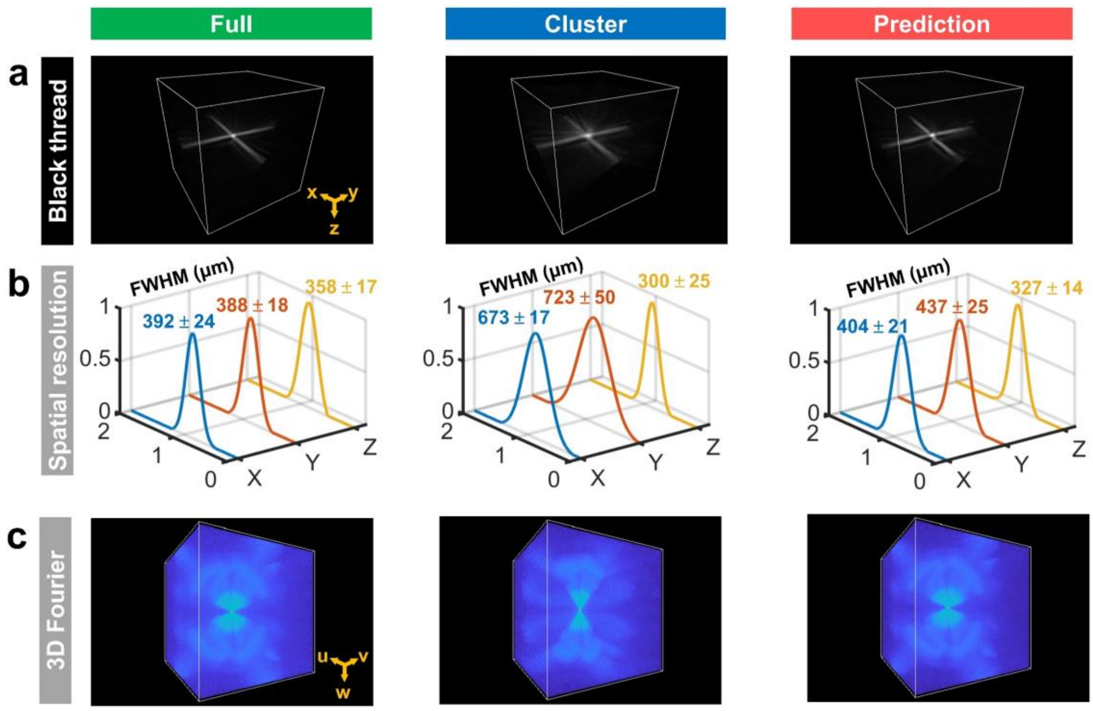

Figure S8.
(a) 3D PA images of a 30-μm carbon fiber, **(b)** spatial resolutions, and **(c)** 3D FFT images in the full, cluster, and DL prediction views.

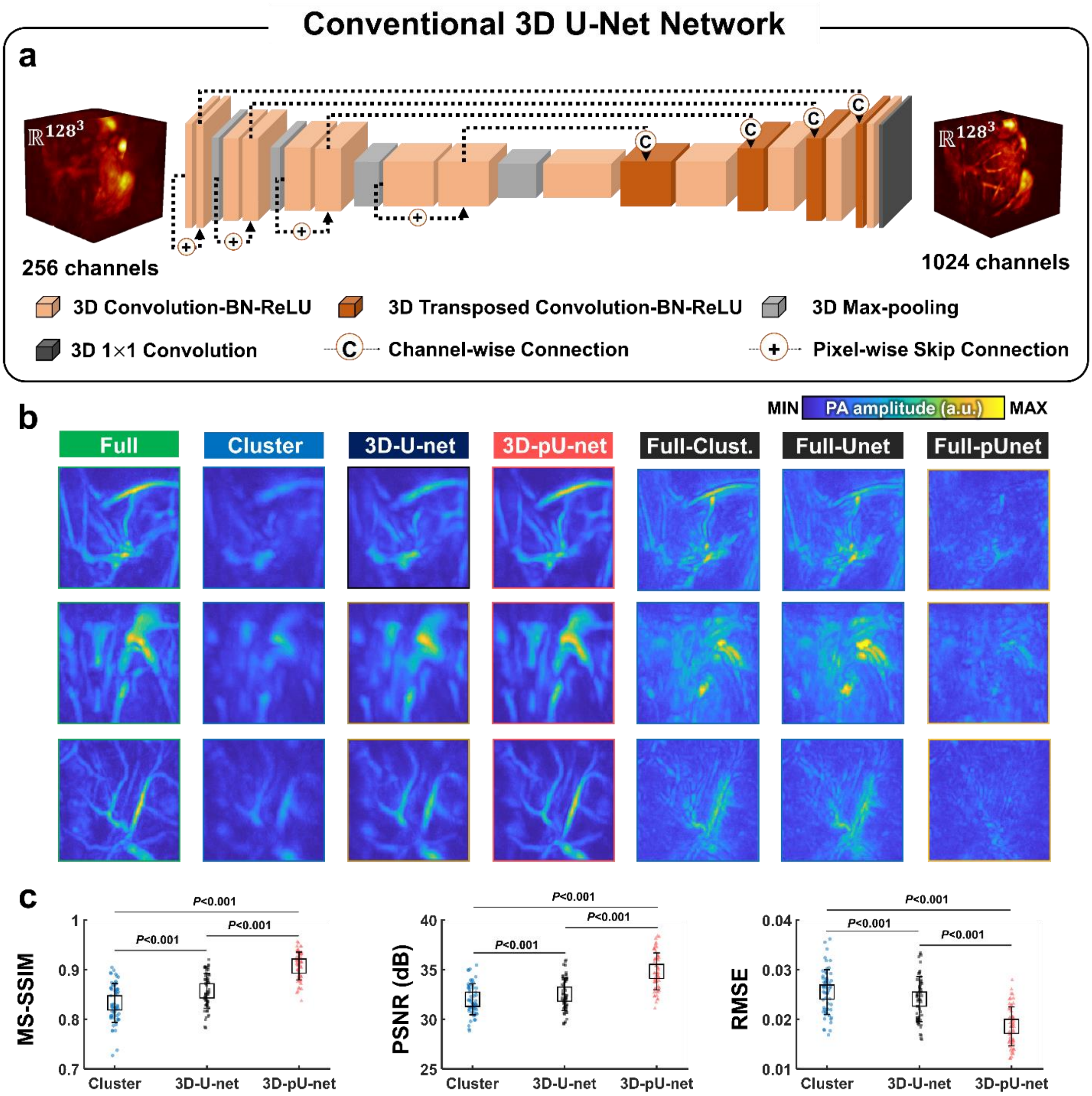

Figure S9.

(a) Architecture of the conventional 3D U-net network. **(b)** Difference in PA amplitudes of the cluster-view, conventional 3D-U-net prediction, and proposed 3D-pU-net prediction images with respect to the full-view image. **(c)** Comparisons of MS-SSIM, PSNR, and RMSE of the cluster-view, conventional 3D-U-net prediction, and proposed 3D-pU-net prediction images with respect to the full-view image.

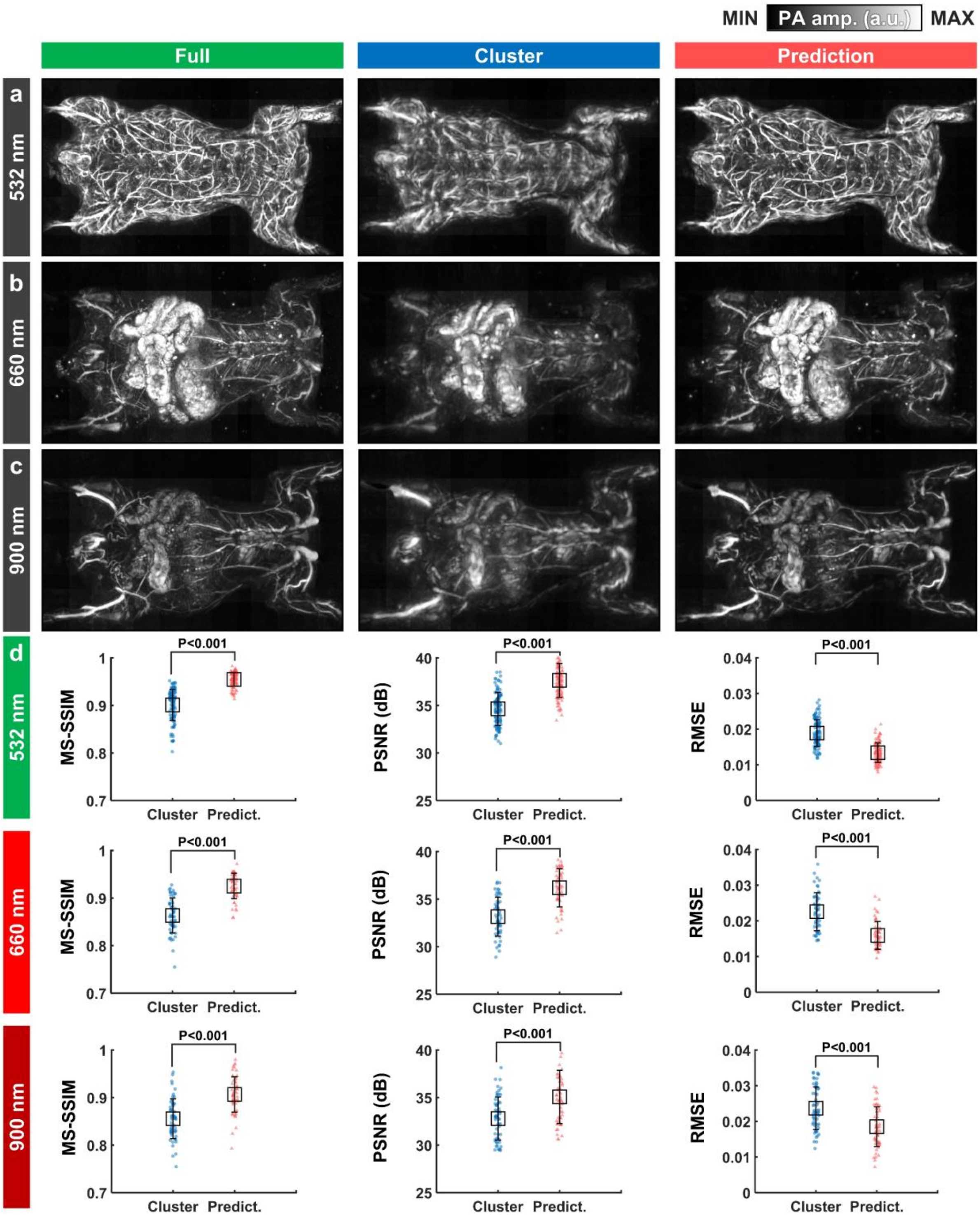

Figure S10.
Whole-body ventral PA MAP images of a rat *in vivo*, showing in the full view, cluster view, and DL prediction at 532 nm, 660 nm, and 900 nm.

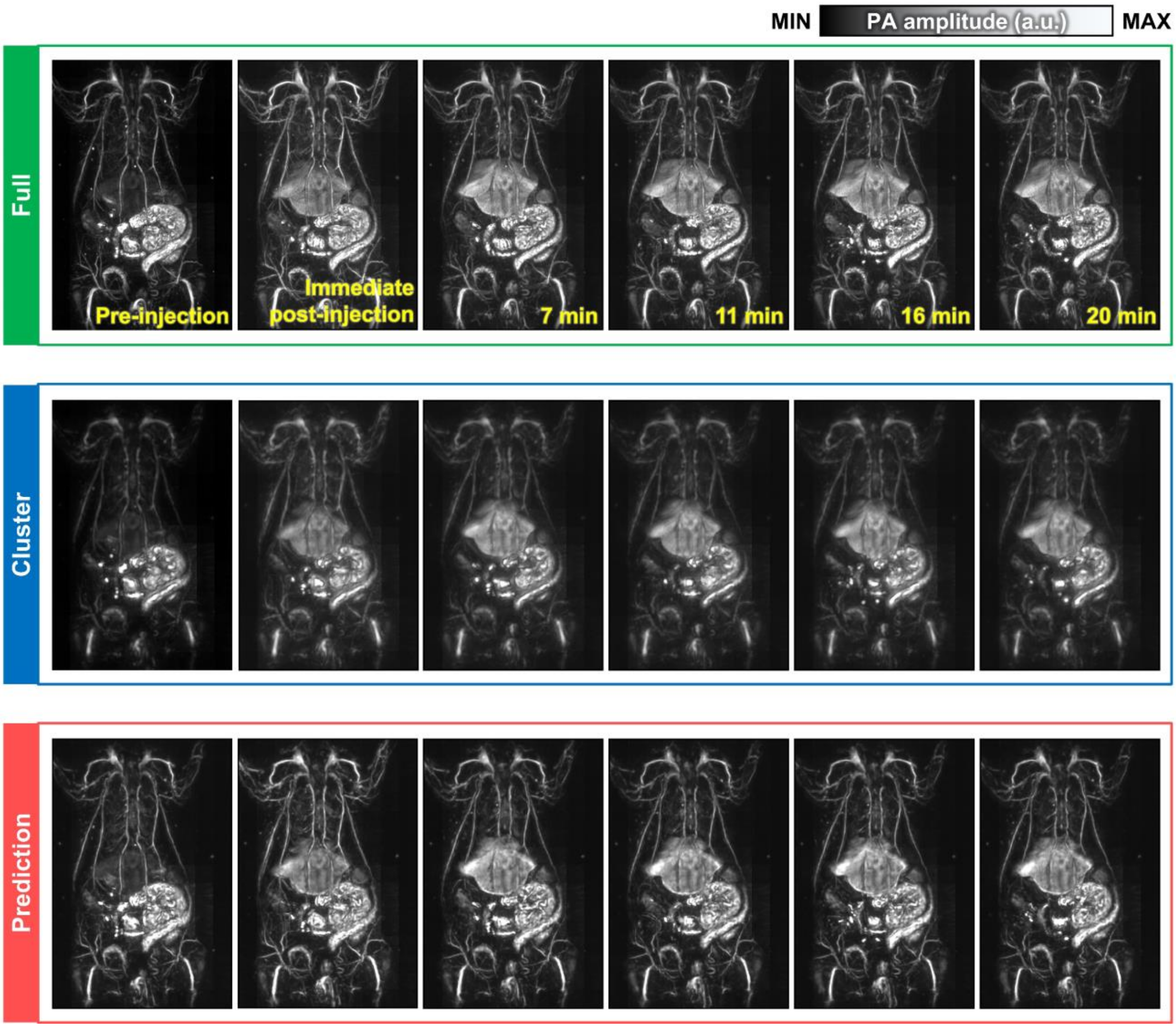

Figure S11.

Whole-body ventral PA MAP images of a rat *in vivo*, showing in the full view, cluster view, and DL prediction under the tail-vein ICG injection.

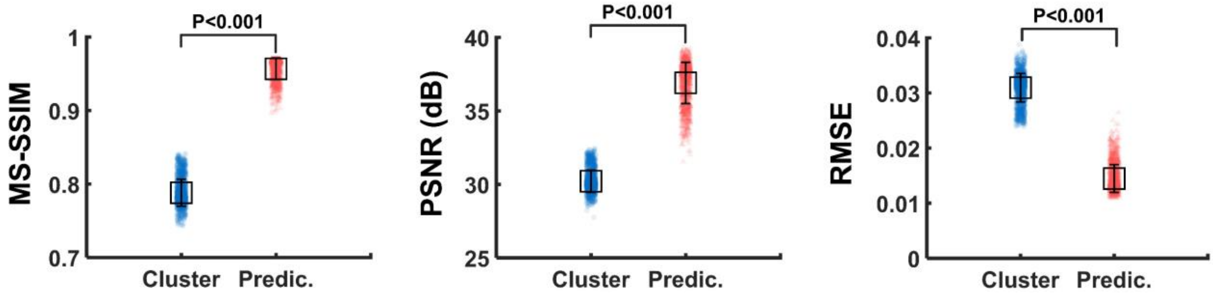

Figure S12.
Comparisons of MS-SSIM, PSNR, and RMSE of the cluster-view or prediction images with respect to the full-view image at all time points in Figure 3b.

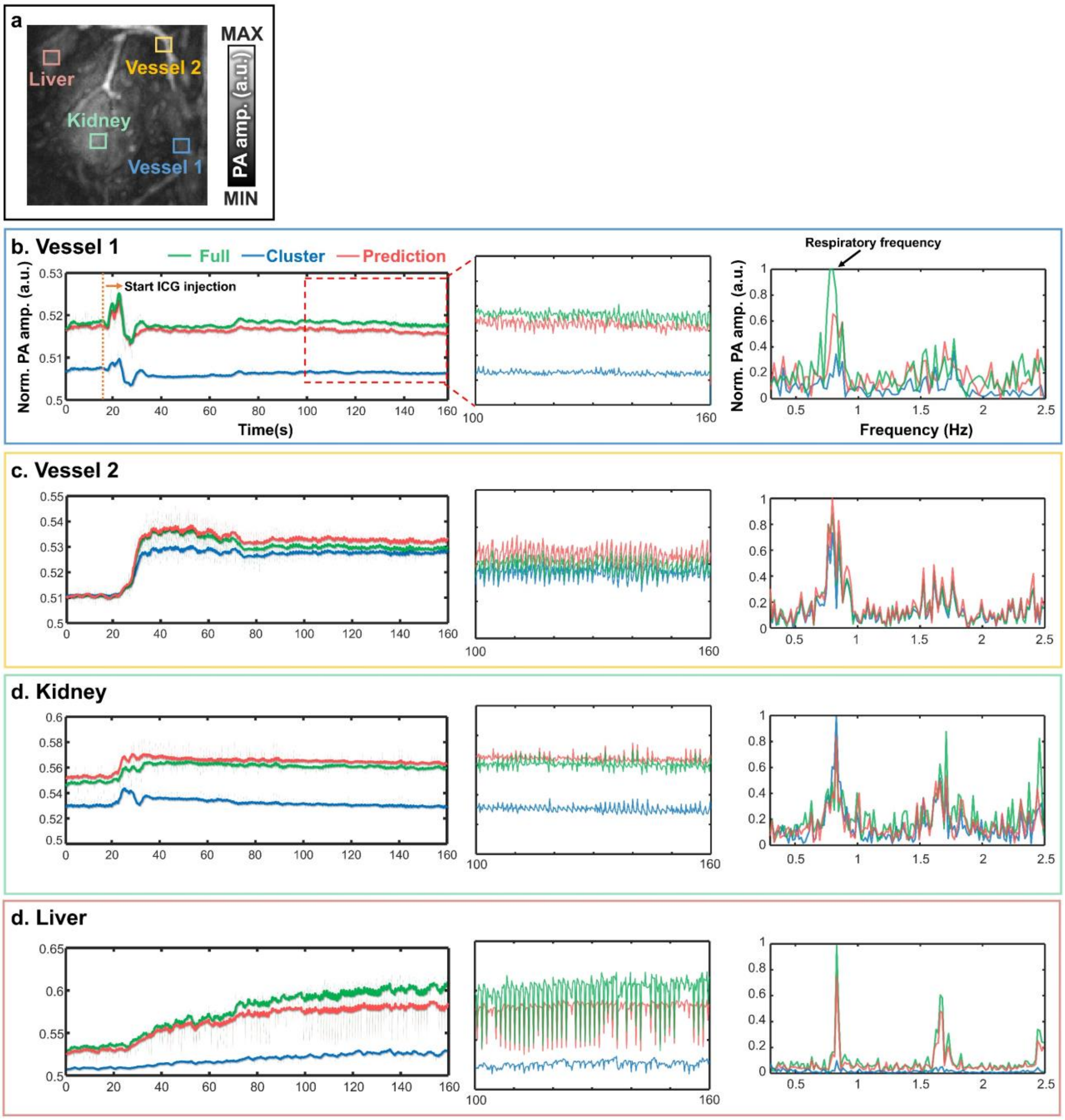

Figure S13.

(a) Close-up PA MAP image of the rat's kidney. Time-lapse PA signal traces and their power spectra for **(b)** vessel 1, **(c)** vessel 2, **(d)** liver, and **(e)** kidney, with the full view, cluster view, and DL prediction.

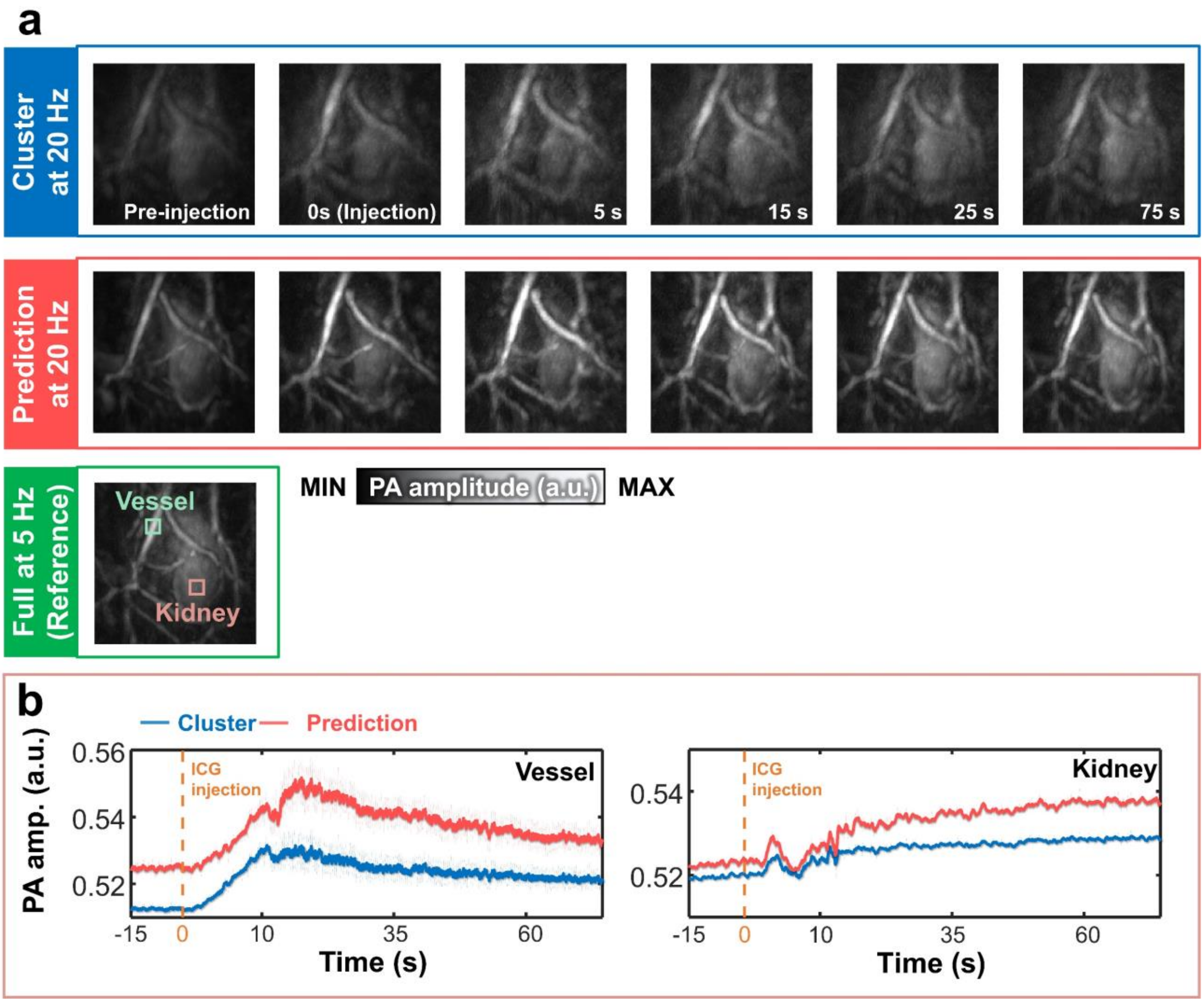

Figure S14.
(a) Dynamic contrast-enhanced cluster-view and DL-predicted PA images following the first ICG injection, acquired at 20 Hz, the maximum imaging speed. Note that there is no ground truth image for the DL prediction. A full-view image is acquired at 5 Hz with the second ICG injection as a reference. (b) Time-lapse PA signals in the vessel and kidney.

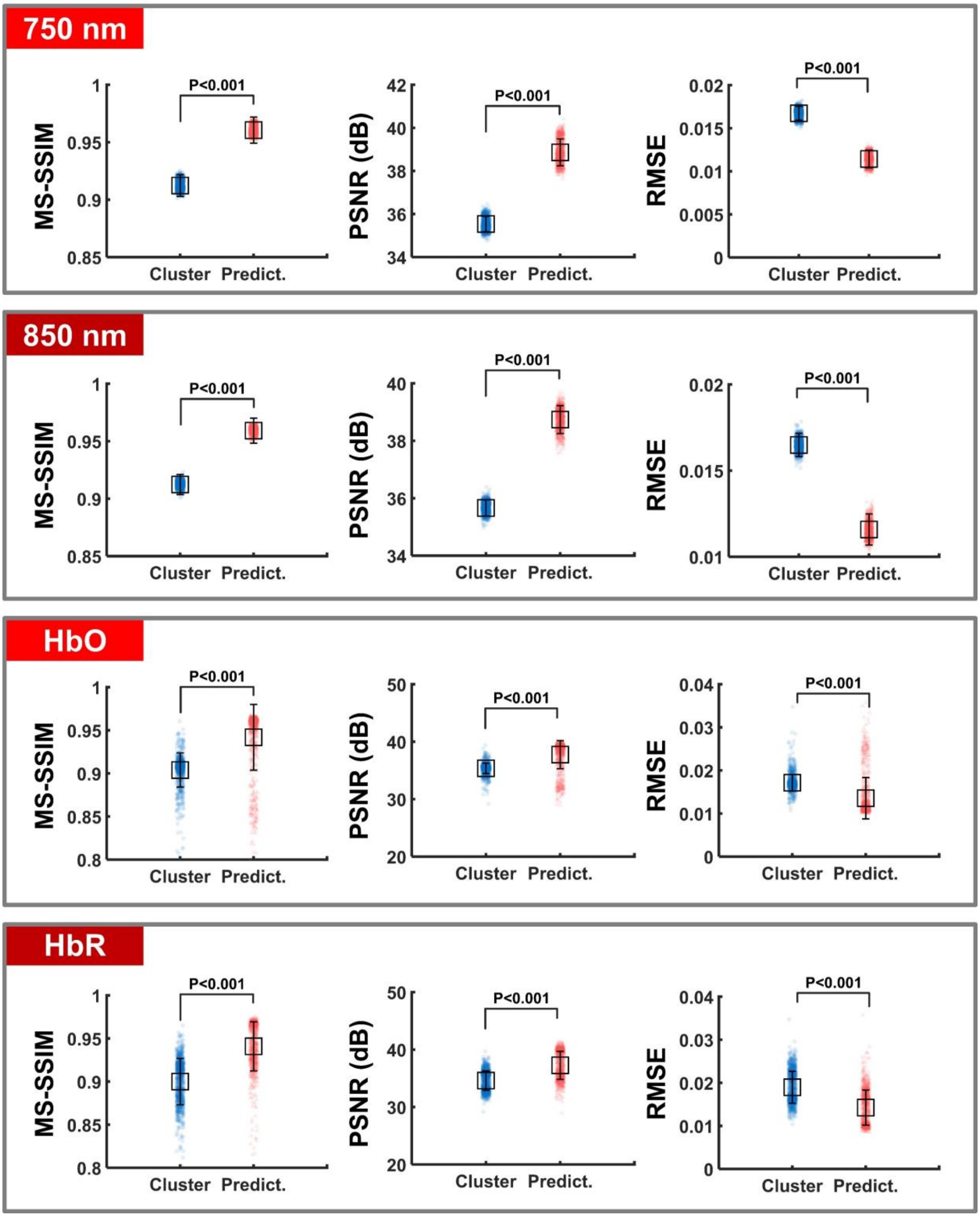

Figure S15.
Comparisons of MS-SSIM, PSNR, and RMSE of the cluster-view and DL prediction 750-nm, 850-nm, HbO and HbR images with respect to the full-view images acquired under the oxygen challenge.

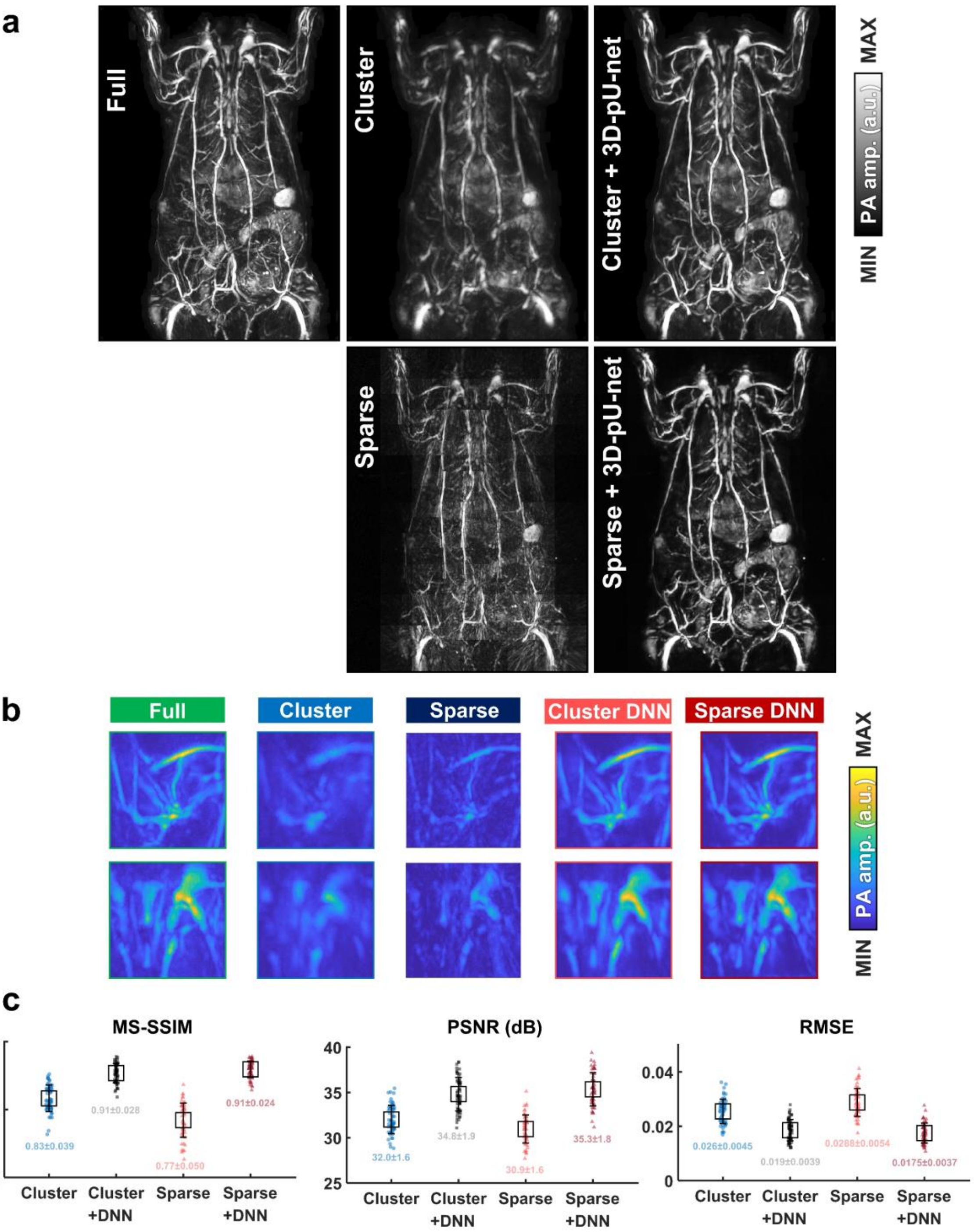

Figure S16. Comparison of the DL prediction with the cluster and sparse data.
(a) Whole-body ventral PA MAP images and **(b)** PA MAP images of a single-volume data of a rat *in vivo*: full view, cluster view, sparse view, DL prediction of cluster view, and DL prediction of sparse view. **(c)** Comparisons of the MS-SSIM, PSNR, and RMSE of the cluster-view, sparse-view, and prediction images with respect to the full-view image.

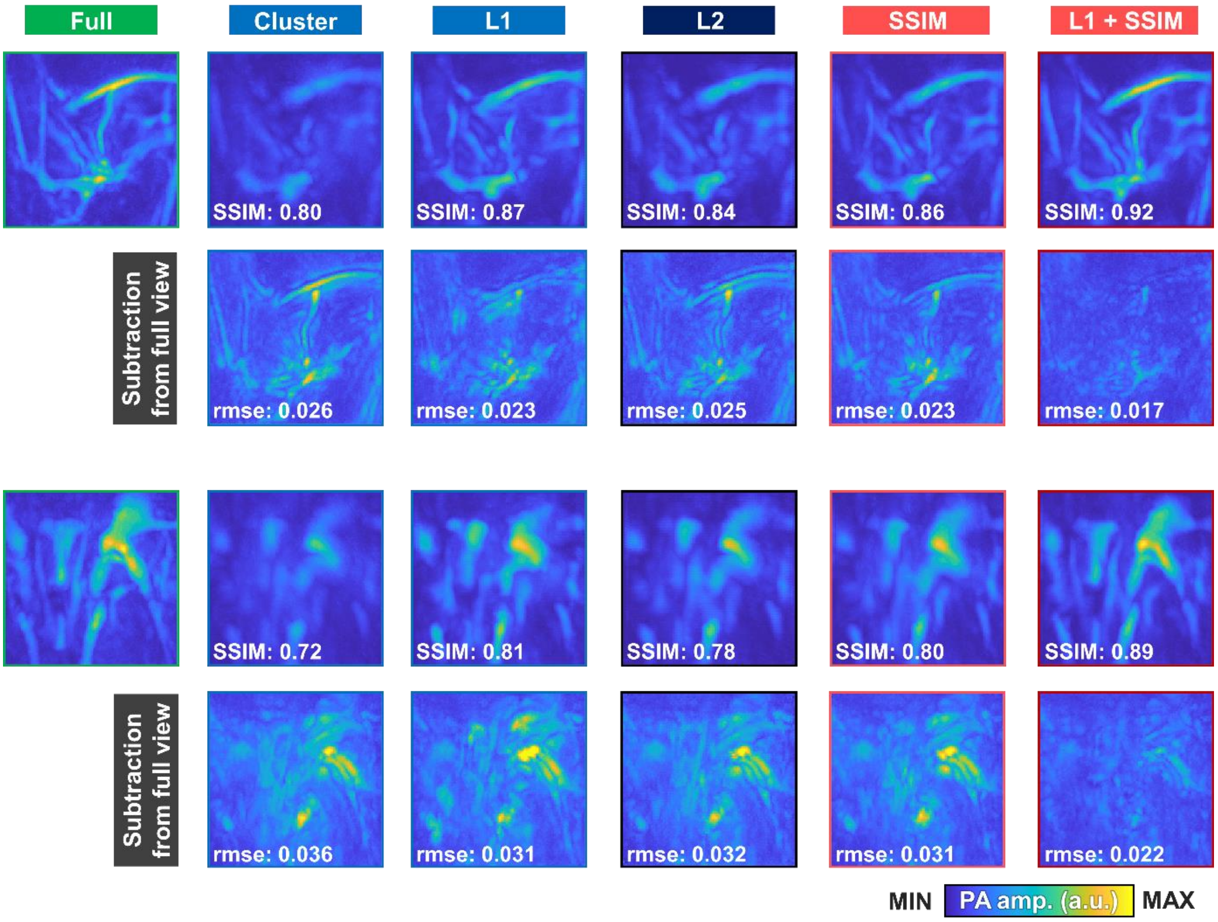

Figure S17.
Comparison of DL prediction among stand-alone and combined loss functions.

Table S1. Interference speed on single GPU

Inference engine	Mini-batch size	Prediction size	Prediction speed* (sec/single volume)
NVIDIA Geforce RTX 3090 (24 GB Memory)	1	128 × 128 × 128 voxels (with a 0.1-mm scale)	$0.026 \pm 1.0e\text{-}4$
	2		$0.024 \pm 3.9e\text{-}5$
	4		$0.023 \pm 2.9e\text{-}5$
	8		$0.022 \pm 2.6e\text{-}5$
	16		$0.018 \pm 8.8e\text{-}6$
	30 (Max. capacity)		$0.016 \pm 3.1e\text{-}5$

*Average 10 runs of evaluation.

Table S2. Data arrangement

	No. of volumes	Subject	No. of subject	Wavelength	Anatomical location
**Training dataset**	1089	Rat	18	900	Ventral/dorsal/sagittal
**Test dataset**					
Static whole body	200	Rat	3	900	Ventral/dorsal/sagittal
Dynamic contrast enhanced whole body	372	Rat	1	800	Ventral
ICG kidney	1500	Rat	1	800	Dorsal
ICG kidney without ground truth	1800	Rat	1	800	Dorsal
sO2 brain	1800	Rat	1	750/850	Dorsal
heart	1200	Rat	1	900	Ventral
Multispectral whole body	252	Rat	1	532/660/900	Ventral
Tumor	40	Mouse	1	866	Sagittal
Blood vessels	63	Human palm	1	800	

Table S3. Hyper-parameter settings

**Hyper-parameter type**	**Description**	**Value**
Training settings	Learning rate	1e-4
	Batch size	[16, 16, 8, 4] *
	Batch normalization momentum	1e-5
	Loss function significance	0.2
Regularization settings	Weight decay coefficient	2e-6
	Reducing patience of learning rate	5
	Reducing factor of learning rate	0.9
	Early stopping patience	15

*Batch sizes of progressive sub-networks are sequentially represented.

Movie Legends

Movie S1. Whole-body ventral, dorsal, and sagittal PA depth-encoded images of a rat, with the full view.

Movie S2. Whole-body ventral PA depth-encoded images of a rat, with the full view, cluster view, and DL prediction.

Movie S3. Time-lapse PA MAP images of the rat's kidney in the full view, cluster view, and prediction.

Movie S4. Time-lapse PA MAP images of the rat's kidney at the 20-Hz imaging speed.

Movie S5. Time-lapse PA sO2 images of the rat's brain under the oxygen challenge in the full view, cluster view, and prediction.

Movie S6. Time-lapse PA MAP images of the rat's heart under at the 20-Hz imaging speed.
